# Curcuminoid-Based Responsive Surfaces for Fluorescent BF_3_ Detection, a Fast and Reversible Approach

**DOI:** 10.1021/acsami.4c19421

**Published:** 2025-03-25

**Authors:** Raquel Gimeno-Muñoz, Raúl Díaz-Torres, Silvia Gómez-Coca, Olivier Roubeau, José Manuel Díaz-Cruz, Núria Aliaga-Alcalde, Arántzazu González-Campo

**Affiliations:** † Institut de Ciència de Materials de Barcelona (ICMAB-CSIC), Campus de La Universitat Autonoma de Barcelona, Barcelona 08193, Spain; ‡ Departament de Química Inorgànica and Institut de Recerca de Química Teòrica I Computacional, Universitat de Barcelona (UB), Diagonal 645, Barcelona 08028, Spain; § Instituto de Nanociencia y Materiales de Aragón (INMA), CSIC and Universidad de Zaragoza, Plaza San Francisco s/n, Zaragoza 50009, Spain; ∥ Departament d’Enginyeria Química I Química Analítica, Universitat de Barcelona (UB), Diagonal 645, Barcelona 08028, Spain; ⊥ ICREA (Institució Catalana de Recerca I Estudis Avançats), Passeig Lluís Companys 23, Barcelona 08010, Spain

**Keywords:** curcuminoids, curcumin derivatives, active materials, fluorescence, chemosensors, responsive surfaces, BF_3_

## Abstract

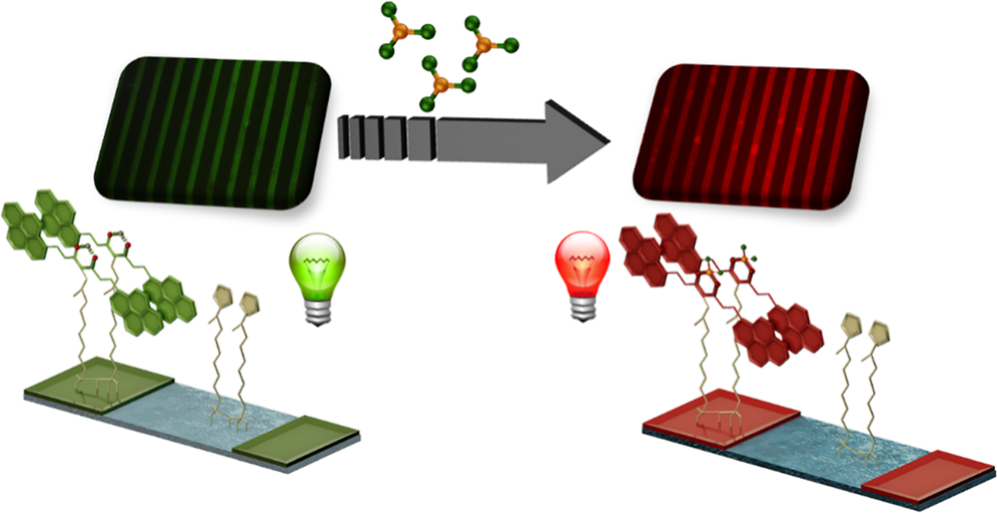

The strategic design of a novel curcuminoid (CCMoid), termed PA, containing pyrene units and a terminal carboxylic group provides the necessary tools for its efficient immobilization on surfaces and its potential use as an optical chemosensor. To this end, our work provides a robust methodology for the preparation of CCMoid-based active surfaces with a fluorescent response and reusability. The covalent immobilization of the CCMoid is obtained by the reaction of the acidic groups of PA and the imidazole ends of the previously functionalized substrates. In this way, fluorescent patterned surfaces of PA, whose emission could be observed in the visible region thanks to the pyrene groups of the CCMoid, were obtained using microcontact printing. In addition, the coordination of BF_3_ molecules (in solution and in gas phase) with the keto–enol moiety of the PAs anchored on the surfaces has been analyzed. The ability of BF_3_ to modify the optical properties of the CCMoids-based surfaces, leading to emissions in the near-IR, has been identified as a fast and reversible process. Such ability is intrinsic to the final coordinated system and not to other boron-based molecules, providing unique response and sensing surfaces.

## Introduction

The growing demand for portable in situ sensing devices underlines the importance of chemical sensors,^1−4^ in leading-edge applications, for environmental monitoring,^5^ in smart buildings,^6^ food screening^7,8^ and biosecurity.^9^ Among them, fluorescent chemosensors are noninvasive, thus eliminating the need for direct sample handling and allowing remote measurements, and are not susceptible to electromagnetic interference, which is a significant advantage over electrochemical detection methodologies.^10^ Ideally, fluorescent sensors respond in a reversible and fast way, depending on the analyte concentrations, and enable its visualization and distribution analysis due to their high spatial and temporal resolution.^11^ These characteristics make them particularly suitable for on-site detection of toxic or hazardous compounds, where such threats require the development of highly sensitive, selective and easy to visualize sensors.^12^

Here we focus on the detection of BF_3_. This molecule is an inorganic chemical found as a colorless gas, which is commercially available in liquid form stabilized in organic solvents.^13^ BF_3_ is highly reactive, commonly used in isomerization,^14^ polymerization,^15^ and esterification reactions,^16^ among others.^17−20^ Its applications have expanded, as it is widely used in the semiconductor industry as a catalyst in chemical vapor deposition processes.^21,22^ It has been established that the degradation of BF_3_ can lead to the formation of highly acidic, corrosive and toxic species that can be released into the environment in possible leaks, so appropriate plans for their rapid detection must be developed.^23,24^ BF_3_ can harm both the ecosystem and the population, causing skin irritation, respiratory toxicity^25^ and even kidney problems^23,25^ at a threshold limit of 1 ppm.^26^ Therefore, the improvement of techniques and methodologies for BF_3_ detection is a subject of interest. In recent years, the development of chemosensors for BF_3_ detection has emerged as an alternative to more sophisticated and expensive techniques. Although most of the new fluorescent probes are capable to detect BF_3_ in solution,^27−29^ for their applicability in industry and gas detection, it is convenient their loading in matrixes or immobilization on substrates. Until now, the most developed strategy has been their loading on paper stripes,^30−34^ and it is still necessary to investigate alternatives for their incorporation in devices.

In solution, curcumin and curcuminoids (CCM and CCMoids: a family of linear organic molecules with a diarylheptanoid skeleton) have demonstrated their capability to coordinate ion species through the 1,3-diketone unit of these systems. This has triggered their applicability in the sensory field for the detection of metallic centers, in biological or industrial processes.^35^ This way, it is known that embedded CCM in different matrices can act as a sensor for heavy metal ions such as Pb(II),^36^ As(III),^37^ Fe(III).^38^ Moreover, CCM and CCMoids have demonstrated rapid binding with BF_3_ species, producing relevant optical changes.^39−42^ Their coordination causes the enhancement and bathochromic shift of their emission bands, due to the formation of donor–acceptor–donor structures.^41,42^

**Figure 1:**
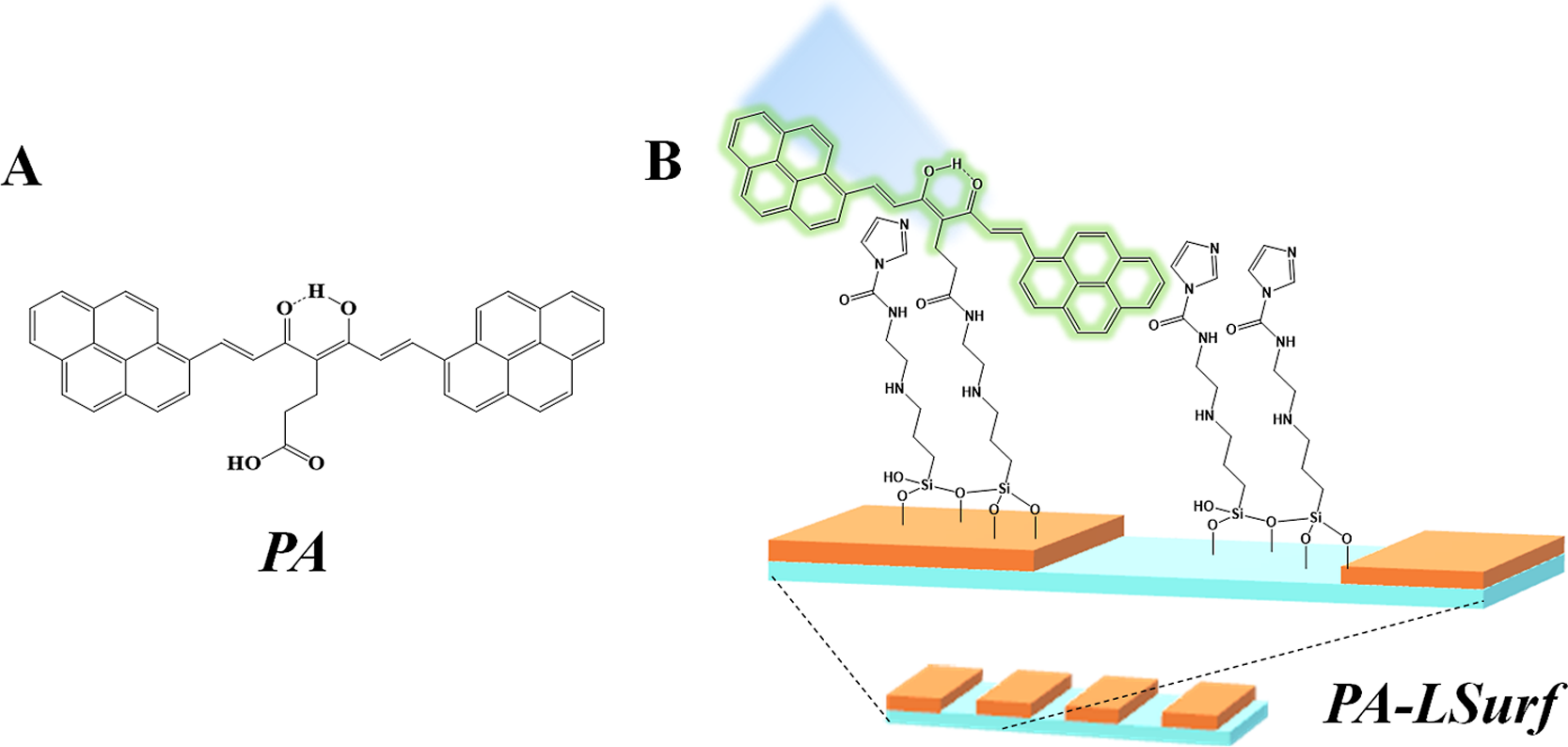
(A) Chemical structure of pyrene based CCMoid, (PA) and (B) PA micropatterned surfaces (PA-LSurf).

Here, driven by the potential of CCMoids, as easily created molecular platforms, together with their ability to coordinate BF_3_, we describe a straightforward strategy to create an efficient, robust, and reusable probe for solution and gas analysis. To this end, we designed a new T-Shape CCMoid with fluorescent properties (PA, Figure 1A) and immobilized it on functionalized Si(100) wafers and glass surfaces (PA-LSurf, Figure 1B). Such combination has resulted in highly reactive surfaces toward BF_3_ with rapid and reversible response.

## Experimental Section

Chemicals and instrumentals used are described in the Supporting Information, together with the synthesis procedures and all the characterization data of the compounds (PE, 4-acetyl-5-oxohexanoic acid, PA and PABF_2_).

### Immobilization of PA on Surfaces (PA-LSurf)

Preparation of polydimethylsiloxane (PDMS) stamps and IM-SAMs^43^ is described in the Supporting Information.

### Printing Process (μCP)

PDMS stamps were inked with a solution of 1 mM PA in DMF for 1 h and dried with a nitrogen stream. It is important to cover them during this time to avoid degradation with light. The stamps were then brought into contact with the IM-SAM, with a weight of 10 g on them, and the whole system was kept in a vacuum desiccator, overnight. The stamps were removed, and the surfaces were rinsed with DMF and dried with a nitrogen stream. (Due to the reactivity of IM-SAMs with water, the printing process was performed with less than 30% of atmospheric humidity conditions).

## Detection of BF_3_·O(C_2_H_5_)_2_ with PA-LSurf (PABF_2_-LSurf)

### PABF_2_-LSurf in Solution

Patterned PA surfaces were immersed in solutions of BF_3_·O(C_2_H_5_)_2_ in dry DCM of different concentrations for 1 min, respectively. After, the surfaces were rinsed with dry DCM and dried with a nitrogen stream.

### PABF_2_-LSurf in Vapor Phase

Patterned PA surfaces were placed in a closed 3D-printed setup,^44,45^ where the surface was placed 3 cm from the bottom of the set containing different volumes (μL) of BF_3_·O(C_2_H_5_)_2_. After 2 min, the surface was removed, washed with dry DCM and dried with a nitrogen stream.

### Reusability of PA-LSurf

A solution of 30 mL of Milli-Q water with NaOH pellets was prepared until the pH reached 12. Subsequently, the PABF_2_-LSurf surface was immersed in the basic solution for 4 min, washed with Milli-Q water, and dried with a nitrogen stream. Finally, the samples were kept in a vacuum desiccator overnight before inspection using a fluorescence microscope.

### General Measurements: Fluorescence Quantification in Digital Imaging

Sample preparation, image acquisition parameters and data processing for fluorescence quantification of the images and measurement of the signal-to-noise ratio (SNR) are detailed in the Supporting Information.^46^

## Results and Discussion

### Synthesis of PA and PA-BF_2_

The synthesis of PA T-Shape CCMoid started with certain modifications to the Pabon method described in the Supporting Information (Scheme S1).^47^ Two synthetic routes were tested for this purpose, yielding PA CCMoid with a yield of 66%. On the other hand, with the objective to study the fluorescent properties of PA upon complexation with BF_3_, the corresponding boron difluoride complex CCMoid (PABF_2_) was also prepared (Scheme S2). The structures of PA and PABF_2_ were confirmed by ^1^H NMR and ^13^C NMR spectroscopy, FTIR-ATR, elemental analysis and MALDI-TOF. Moreover, the crystal structure of PE CCMoid was also resolved (See Supporting Information, Figures S1–S9, Tables S1 and S2), confirming the molecular structure of this intermediated.

## Optical Properties of PA and PABF_2_

**Figure 2:**
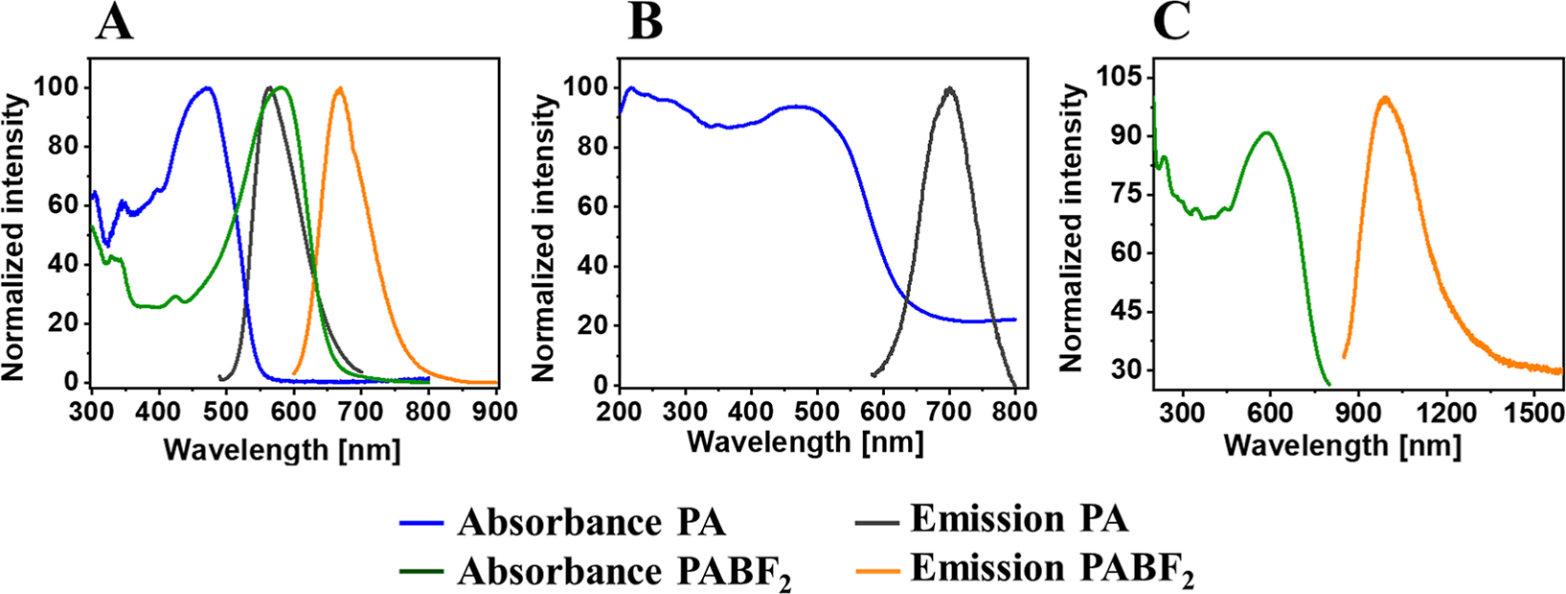
(A) Normalized absorbance and fluorescence emission spectra of PA and PABF_2_ in DCM at 10^–5^ M; (B) absorbance and fluorescence in solid state of PA and (C) PABF_2_ CCMoids.

The electronic UV–visible absorption and fluorescence emission spectra of PA and PABF_2_ were recorded in DCM solution using a concentration of 10^–5^ M, Figure 2A. The maximum of the PA absorption band appeared at λ_max_ = 480 nm (blue line), related to the typical π–π* transition of CCMoids keto–enol system; in addition, bands at 395, 347, and 305 nm also appear referring to the π–π* transitions of the pyrenes groups.^48,49^ For PABF_2_ the maximum absorption band appeared at λ_max_ = 586 nm (green line), and the rest of the bands related to the pyrene groups at 424, 339, and 299 nm.

The fluorescence behaviors of both systems are also shown in Figure 2A. To study them, the PA solution was excited with a λ_exc_ of 485 nm, which produced the observation of an emission band that showed a maximum peak at λ_em_ = 563 nm (green-yellow region, in the range of 490–580 nm, gray line). Upon excitation of the PABF_2_ compound with a λ_exc_ of 587 nm, an emission band with a maximum peak at λ_em_ = 668 nm was observed in the red-shift area (range 650–800 nm, orange line). Although PA and PABF_2_ presented the same number of transitions, in the case of PABF_2_, there was a dramatic bathochromic change (>100 nm difference) caused by the coordination with the -BF_2_ group, which, together with the presence of the pyrenes, gave the molecule a D-π–*A*–π–D type structure. In addition, the incorporation of the -BF_2_ group is known to increase the rigidity of the molecule and improve its photostability.^42,50^ Similarly, UV–vis absorbance characterization of PA and PABF_2_ in the solid state was also performed, owing to the solvochromic character of most CCMoids in solution (Figure 2B,C). For that, samples were prepared with pellets of KBr having 0.03 mg of the respective CCMoids. The maximum absorption band of PA appeared at λ_max_ = 495 nm (green region, Figure 2B, blue line), whereas that of PABF_2_ occurred at λ_max_ = 601 nm (orange region, Figure 2C, green line). Overall, the absorbance bands shifted by 10–20 nm, toward larger wavelength values, compared with those obtained in DCM.

In addition, the solid state fluorescence emission of PA was studied, exciting the sample at λ_exc_ = 485 nm detecting an emission band at λ_em_ = 700 nm (red region, Figure 2B, dark gray line). Similarly, the emission of PABF_2_ in the solid state was recorded by exciting the sample at λ_exc_ = 600 nm, and an emission band was observed at λ_max_ = 981 nm (within the NIR zone, Figure 2C, orange line).

Therefore, both systems displayed absorbance and emission bands that showed clear differences in the optical properties in the solid state as well as in solution. Moreover, it is important to stress that PA in solution showed emission within the green range, and in comparison with the solid state, the latter showed a shift of approximately 200 nm toward wavelengths of the near-infrared zone. Here, coordination to the -BF_2_ moiety and intermolecular interactions may combine to provide great displacements and therefore changes in the optical properties. Studies of CCMoids coordinated to -BF_2_ describe similar behavior in the solid state and propose mechanisms for the redshift based on a variety of possible CCMoid aggregations.^51,52^ Furthermore, the quantum yield (Φ) calculated for a 10^–4^ M solution of PA in DCM was 0.16 ± 0.02, 1 order of magnitude larger than a similar CCMoid containing anthracene groups.^53^ Therefore, the design of the PA CCMoid aims to improve its emission in the presence of pyrene groups.

### PA Funcionalized Surfaces (PA-LSurf)

**Figure 3:**
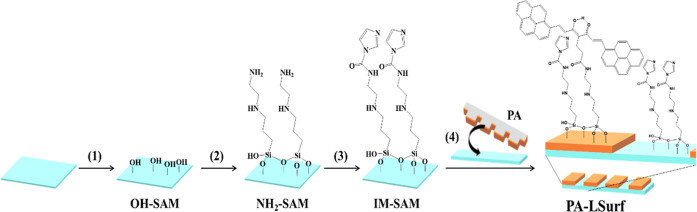
Preparation of PA-functionalized surfaces: (1) activation of the surface with piranha and a basic solution, (2) functionalization in vapor phase with TPEDA overnight at 70 °C (NH_2_–SAM), (3) immersion of the surfaces in saturated CDI solution with dry THF for 4 h under Ar (IM-SAM), and (4) immobilization of the PA molecule on IM-SAM by printing (μCP) to obtain PA-LSurf.

The immobilization of the PA on surfaces was performed by microconctact printing (μCP), as schematized in Figure 3. First, an imidazole-terminated monolayer (IM-SAM)^43^ was prepared and its formation was confirmed by contact angle measurements, fluorescence microscopy and X-ray photoelectron spectroscopy (XPS) (Figures S10–S12). Then, PDMS stamps were employed to form either patterned (stamps with lines) or full (stamps without features) monolayers of PA. The oxidized stamps were inked with a solution of PA in DMF and placed in contact with the surfaces. This solvent completely dissolves PA and is compatible with PDMS stamps.^54^ Due to the contact between the stamps and the IM-SAMs, the carboxylic acid of PA reacted with the terminal imidazole group on the surface, forming an amide bond, resulting in patterned PA monolayers or full PA functionalized surfaces depending on the stamp used, with and without motifs, respectively.

**Figure 4:**
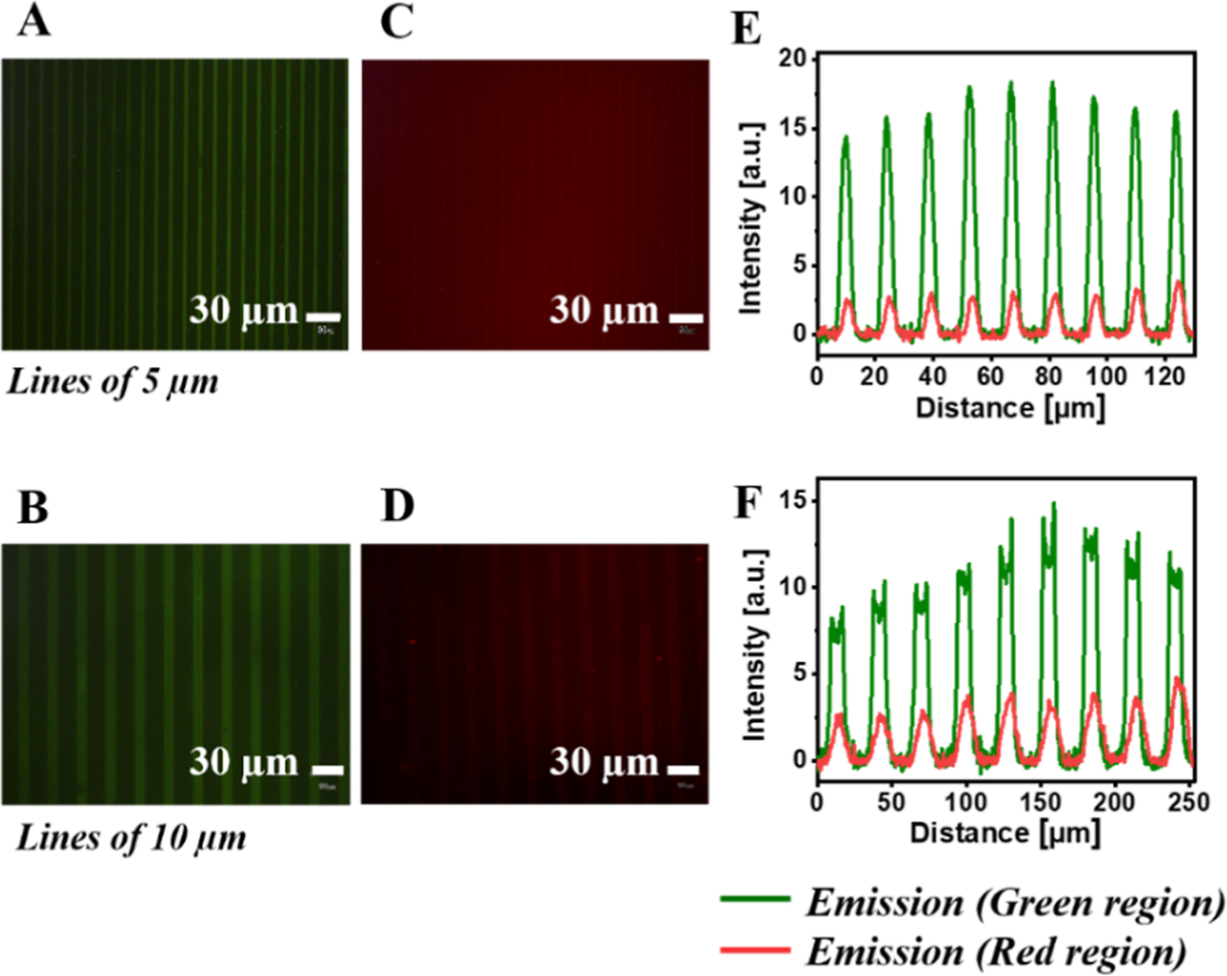
Fluorescent microscopy images of micropattern of PA lines on a glass surface (PA-LSurf) and intensity profiles. Conditions: low humidity, oxidized PDMS stamps 1 min with O_2_ plasma, inking 1 h with 1 mM PA and μCP overnight. Images obtained by fluorescence microscopy (×20 magnification, aperture 8). (A) 5 μm and (B) 10 μm lines with emission in the green region (filter 450 nm < λex <480 nm; λem ≥515 nm) and (C) 5 μm and (D) 10 μm lines with emission in the red region (filter 510 nm ≤ λex ≤550 nm; λem ≥590 nm). Intensity plot profile of micropatterns of lines of (E) 5 μm and (F) 10 μm.

After removing the stamp and washing the surfaces, the resulting line patterns of PA-LSurf on glass slides were inspected by fluorescence microscopy, which also allowed the optimization of several parameters of the printing process, such as the concentration of the PA ink solution, printing time, oxidation of the stamps, and the influence of atmospheric humidity.^55^ The fluorescent patterns of PA clearly showed a sharp, uniform contrast between the CCMoid areas and the background when a blue filter (450 nm < λ_ex_ < 480 nm; λ_em_ ≥ 515 nm) was used (Figure 4A,B,E,F).

When the surface was irradiated and a green filter was used (510 nm ≤ λ_ex_ ≤ 550 nm; λ_em_ ≥ 590 nm), almost no patterns were detected (Figure 4C–F). In the latter, the low intensity existing is due to the fact that the surface PA emits in a wide range from 450 to 650 nm, with a maximum peak at 408 nm, and a small part of the emission is still collected. Nevertheless, this fact was considered negligible compared to the subsequent response of the surfaces.

In order to achieve the optimal printing conditions, a series of printing experiments were performed modifying the ink concentration and the printing time (see Supporting Information, Figures S13 and S14). First, the better results were obtained with 10^–3^ M inking solution concentration, showing homogeneous patterns and without aggregates (Figure S13). On the other hand, for the printing time optimization study, the surfaces were stored in a vacuum desiccator during the printing to regulate the humidity and prevent degradation of the terminal imidazole groups of the IM SAMs. Although, after 3 h of printing, patterns were observed with the maximum intensity, longer printing times allowed more homogeneous and reproducible patterns with good contrasts (Figure S14).

### Characterization of PA-LSurf

**Figure 5:**
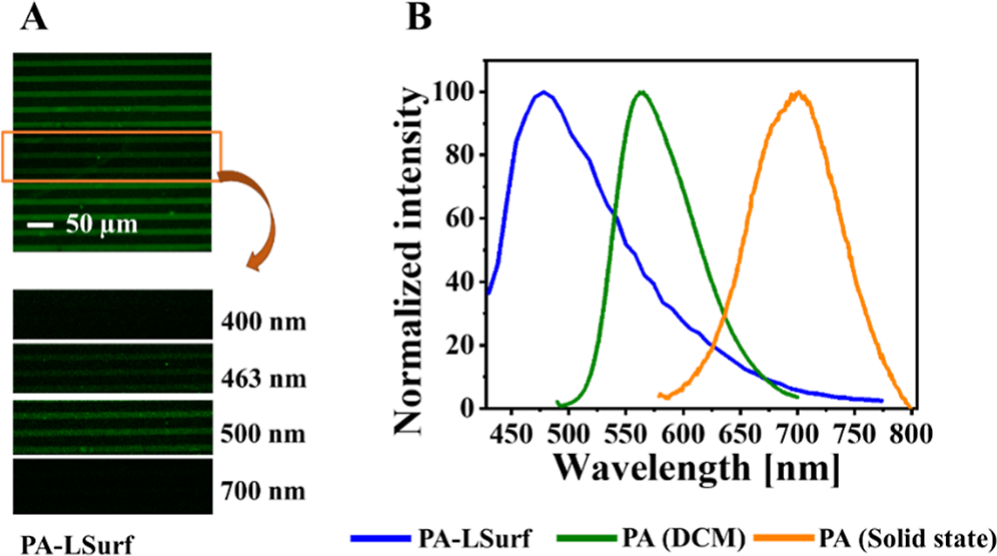
Study of the fluorescence emission of PA on the surface (PA-LSurf) using confocal microscopy. (A) Image of the PA-LSurf study area. (B) Normalized emission spectrum of PA-LSurf (blue), PA in solution (green), and PA in solid state (orange).

The fluorescence emission of the PA-LSurf glass surfaces and their stability were monitored by confocal microscopy. For this, a selected area of the surface was excited at 405 nm. After scanning, the emission spectrum showed a broad band from 440 to 750 nm. with a maximum signal at approximately 476 nm (Figure 5A,B (blue line)).

The comparison of the optical properties of the PA-LSurf with those of PA CCMoid in solution (Figure 5, green line) and in the solid state (Figure 5, orange line) clearly showed how the environment has a great effect on the final emission. To some extent, all CCMoids are solvochromic compounds, while in the solid state (bulk), aggregates are formed displaying a broad emission band in the NIR. PA-LSurf showed a significant shift of the emission of the PA toward much lower wavelengths. Such displacement, which is even lower to that of PA in solution (DCM), it is closer to the emission observed for pyrene monomers and relates to the distribution of PA on the surface.^49,56,57^ This would reinforce the idea of the absence of aggregates or the formation of compact layers that could display strong intermolecular interactions between the CCMoids.

**Figure 6:**
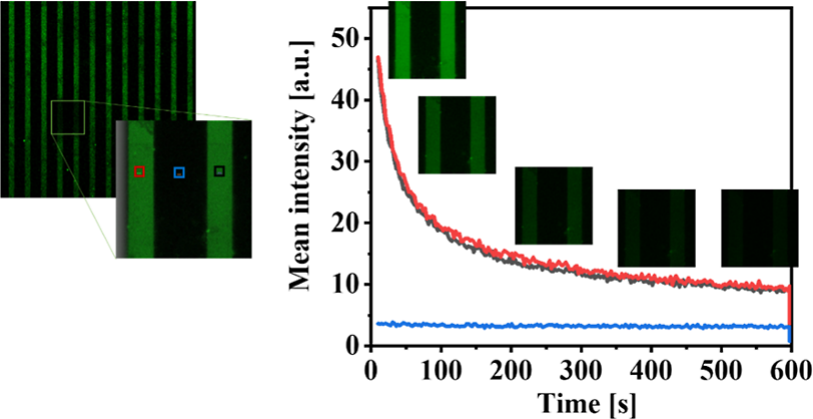
Study of the extinction of PA emission in PA-LSurf, using confocal microscopy. Image of the study area and graph of the intensity over the irradiation time of the sample.

In addition, the stability of the PA-LSurf surface over time was examined in two ways. First, a fresh PA-LSurf surface was prepared, and an emission image was taken. Then, it was stored under nitrogen and shielded from light for two weeks. No intensity loss was observed afterward, proving that this storage method was effective (Figure S15). Second, the extinction time of the PA-LSurf emission was studied when it was continuously irradiated by a laser applying 100% of its power, causing photobleaching of the sample. As shown in Figure 6, the emission faded exponentially after 600 s, probably due to the photon-induced chemical damage of the pyrene groups of the system. This indicates that the degradation of the surface requires long irradiation times when using a powerful laser.

The formation of PA-LSurf was additionally confirmed using contact angle measurements and X-ray photoelectron spectroscopy measurements; see Supporting Information (Figures S10 and S12).

### PA-LSurf as a BF_3_ Detector

**Figure 7:**
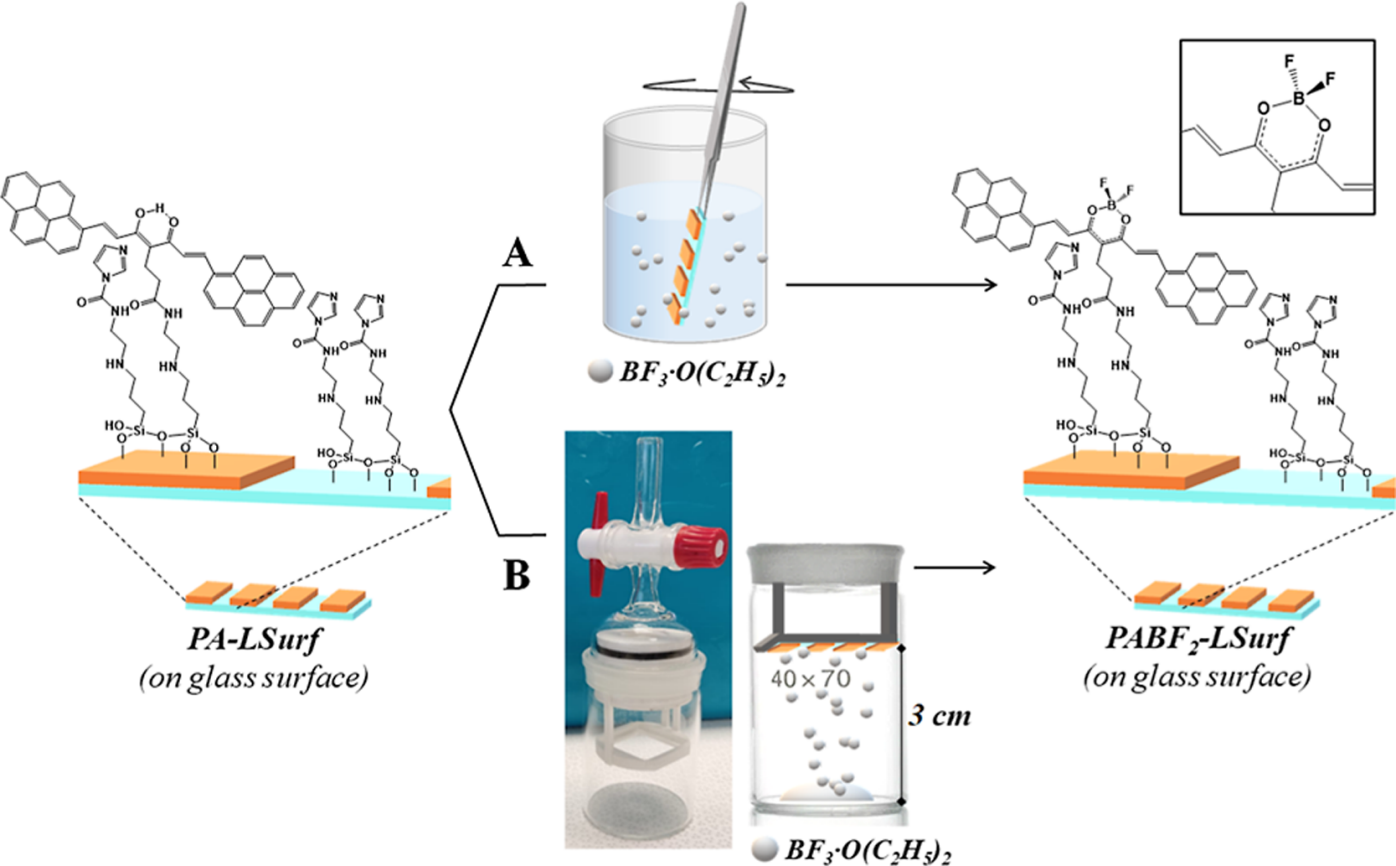
BF_3_ detection methodologies with PA-LSurf by (A) immersion in solution or (B) exposure to BF_3_·O(C_2_H_5_)_2_ vapors.

Once the PA-LSurf was fully characterized, to assess its feasibility for the detection of BF_3_ in solution and in gas phase two approaches were followed: (1) the immersion of the PA-LSurf surface in solution with different concentrations of BF_3_·O(C_2_H_5_)_2_, in dry DCM, to prevent rapid degradation of BF_3_ (Figure 7A) and (2) the exposure of PA-LSurf to the vapors generated by BF_3_·O(C_2_H_5_)_2_ (Figure 7B). Vapor-phase tests were performed using a homemade sublimation setup, with it has possible to perform a small vacuum at first and to keep later the experiment close for short periods of time.^44,45^

Upon exposure of PA-LSurf to BF_3_·O(C_2_H_5_)_2_ a change in the emission of the PA patterns was observed in both methodologies, demonstrating the detection capacity of the fluorescence surface. The reaction between the keto–enol group of immobilized PA CCMoids and BF_3_ is a fast coordination process, resulting in the formation of BF_2_–CCMoid adducts on the surfaces (PABF_2_-LSurf), and a significant shift in wavelengths toward NIR.^50^

### Detection of BF_3_ in Solution by Immersion of the PA-LSurf

**Figure 8:**
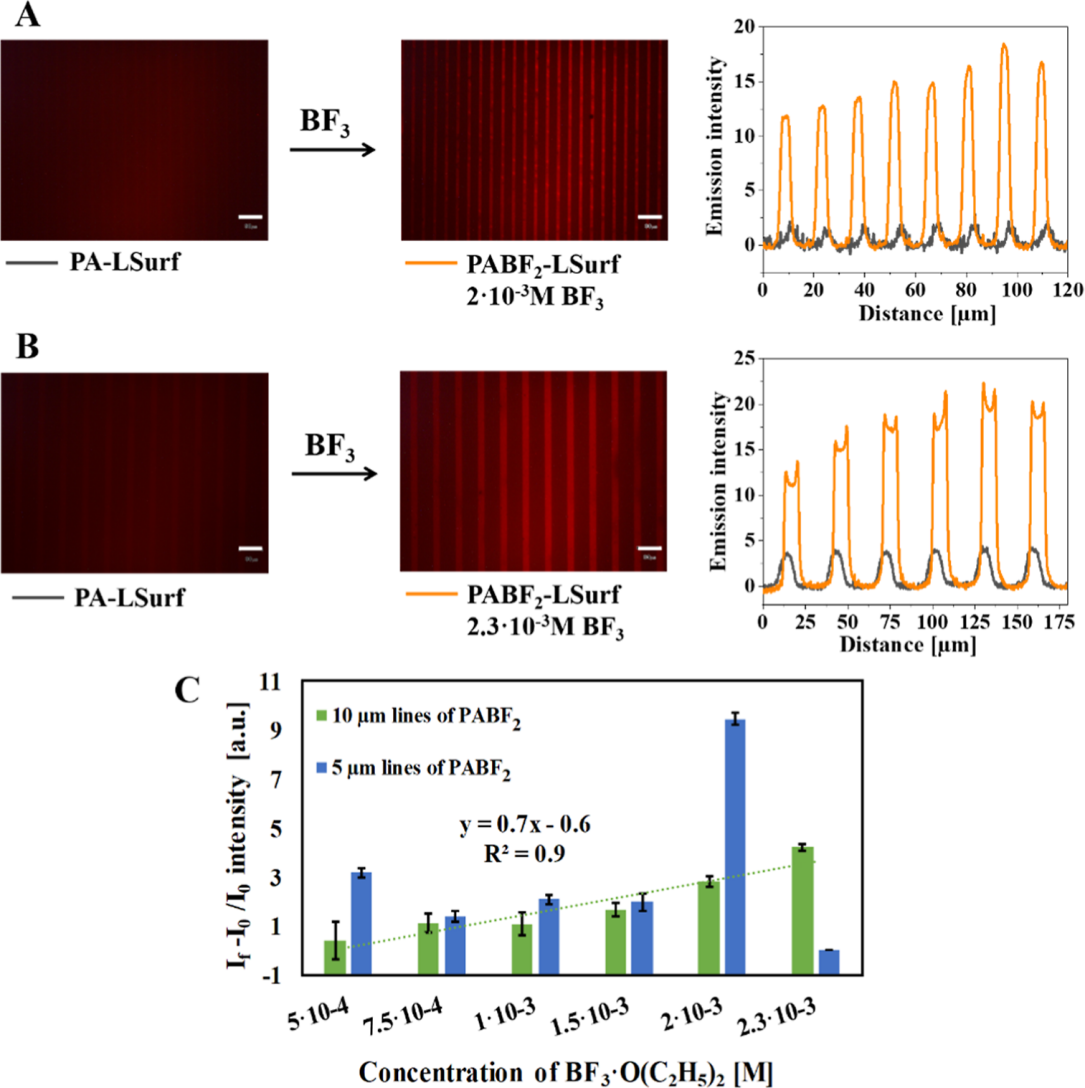
Fluorescence microscopy images of PA-LSurf surfaces reacting with BF_3_ in solution (2 × 10^–3^ M BF_3_ in DCM, 1 min immersion). (A) patterns of 5 μm, while in (B) patterns of 10 μm lines. The plot profile of the initial surface is shown in gray (PA-LSurf) and after the reaction with BF_3_ in orange (PABF_2_-LSurf). (C) Relationship between (*I*_f_ - *I*_0_)/*I*_0_ and BF_3_ concentrations. The intensities of the 5 and 10 μm lines of PA-LSurf with the standard deviations were compared after being immersed for 1 min in solutions with varying concentrations of BF_3_·O(C_2_H_5_)_2_ in dry DCM. The images were acquired using a filter with 510 nm ≤ λex ≤550 nm and λem ≥590 nm, ×20 magnification and aperture 8. Scale bar indicates 30 μm.

To determine the viability of the PA-LSurf system and the detection limit using the immersion methodology, several tests were carried out with different concentrations of BF_3_, ranging from 2.5 × 10^–4^ M to 2.3 × 10^–3^ M of BF_3_·O(C_2_H_5_)_2_ in dry DCM as solvent. The BF_3_ detection study in the solution was limited to a PA-LSurf immersion time of 1 min, as shown in Figure 8.

Long periods of immersion promoted corrosion of the glass surface, probably due to the attack of HF formed as byproduct.^58^ A blank consisting of immersing PA-LSurf in dry DCM for 1 min was made, verifying that the emission intensity of the PA lines remained constant and that the applied conditions unaffected the stability of the monolayer (Figure S16). All tests were performed on surfaces with micropatterns of 5 and 10 μm PA lines, correspondingly, to study the relation between the compactness of the patterns in the response of the surfaces. After performing all tests, the changes in the emission of the patterns were quantified by analyzing the increase in fluorescence intensity and the signal-noise rate (SNR) in the red region (detailed in Supporting Information, Figure S17). In both 5 and 10 μm PA line patterns, changes in intensity were detected analyzing the profiles of the plots from a concentration of 5 × 10^–4^ M of BF_3_, founding an increase in the intensity of 3.20 ± 0.19 a.u. (SNR 4), and 0.41 ± 0.75 a.u. (SNR = 7), respectively (Figure 8C and Table S3).

The improvement in the SNR value with the 10 μm lines could be related to a lower initial noise, which implies a better signal, probably due to a greater amount of PA immobilized on the surface and homogeneous distribution along the lines. Indeed, the micropattern of the 5 μm lines displayed higher variations in the analyzed data, as it can be seen in Figure 8C, while the 10 μm lines showed an increase in fluorescence with a well-defined linear trend with increasing the BF_3_ concentration. Therefore, under the conditions studied, the patterns with 10 μm lines demonstrated higher stability, presenting a good quality of response and very good reproducibility.

Detection of BF_3_ by exposure of PA-LSurf to BF_3_·O(C_2_H_5_)_2_ vapors.

**Figure 9:**
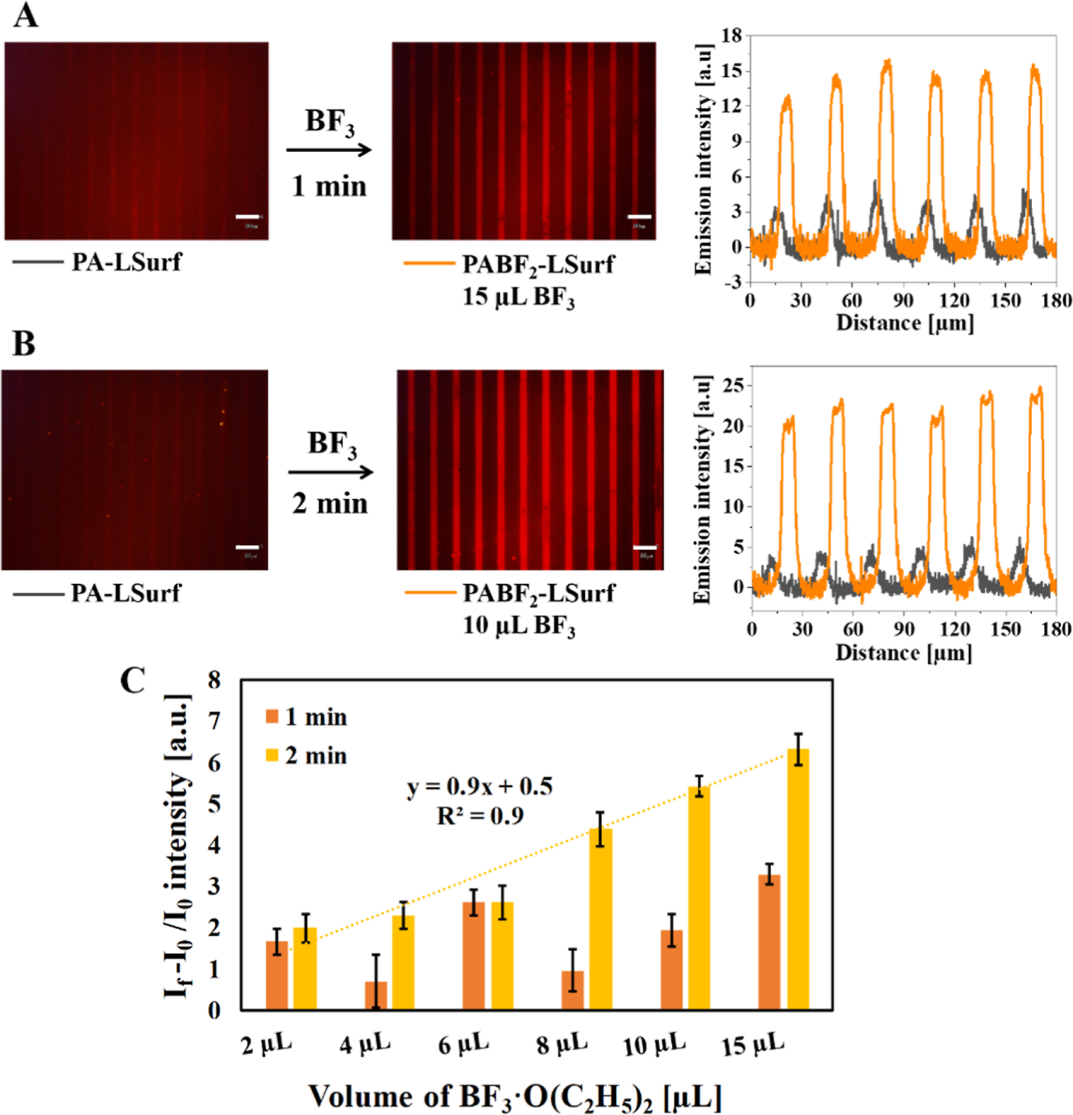
Fluorescence microscopy images of PA-LSurf surfaces exposed to BF_3_ vapors at 25 °C. In (A), an exposure time of 1 min to 15 μL of BF_3_·O(C_2_H_5_)_2_ was used, whereas in (B), 2 min of exposure to 10 μL of BF_3_·O(C_2_H_5_)_2_ was used. The surfaces were kept at a distance of 3 cm from the BF_3_ source. (C) Relationship between (*I*_f_ - *I*_0_)/*I*_0_ and BF_3_·O(C_2_H_5_)_2_ volumes. The intensities of the 10 μm lines of PA-LSurf were compared after exposure to BF_3_·O(C_2_H_5_)_2_ vapors for 1 and 2 min at 25 °C. Standard deviations are also shown. The images were acquired using a filter with 510 nm ≤ λex ≤550 nm and λem ≥590 nm, ×20 magnification and aperture 8. Scale bar indicates 30 μm.

Due to the better response of the PA-LSurf with 10 μm micropatterns, the BF_3_ vapor exposition tests were carried out only with these surfaces. For that, the devices used consisted of two pieces of glassware assembled together, with a set of Teflon supports, where the surface was installed at a fixed distance of 3 cm to different volumes of BF_3_ (in the range 1–15 μL of BF_3_·O(C_2_H_5_)_2_). This system, stabilized with diethyl ether, formed an atmosphere of BF_3_ vapors when the functionalized surface was exposed for 1 or 2 min at 25 °C (Figure 9).

The blank was then prepared using only 15 μL of diethyl ether to determine the stability of the monolayer under these conditions and for comparison reasons. After 2 min of exposure, a decrease in intensity, of approximately 4 a.u., was observed in the PA-LSurf, in the red emission region (Figure S18), but it was considered insignificant, considering that our functionalized substrate was still performing.

The exposition of the PA-LSurf to BF_3_·O(C_2_H_5_)_2_ during 1 min, showed that the emission produced for the PABF_2_-LSurf lines displayed an increase of 1.7 ± 0.3 a.u in intensity in the red region, by analyzing the plot profile of the lines when 2 μL were introduced, Figure 9C. However, from 6 μL onward, it was possible to detect an important change in intensity. Under these conditions, the intensity increased by 2.6 ± 0.3 a.u., and the images were acquired with an SNR of 7, reaching the best SNR of 13 when 15 μL of BF_3_ were employed, with an intensity increase of 3.3 ± 0.2 a.u., Figure 9C and Table S4. In this time frame, the experiments indicated low precision and reproducibility among the tests, concluding that 1 min exposure was not satisfactory; this could relate to the device as well and the lack of a homogeneous atmosphere of BF_3_ in all the experiments.

For that, the exposition time was increased to 2 min, where the fluorescence intensity exhibited a linear relationship with the volume used, indicating the robustness of the system and the formation of the adducts on the surfaces, as it is shown in Figure 9C and Table S4. Therefore, it was considered that the optimal time for the test was 2 min. Under these conditions, the increase was detectable from 2 μL, by analyzing the plot profile, with an increase of 1.9 ± 0.3 a.u, observable with the fluorescence images from 10 μL with an increase of 5.4 ± 0.2 a.u., and an SNR ratio of 18.

Similar to the tests at 1 min, the greatest increase in the intensity was observed at the highest volume tested (15 μL). In this case, after 2 min of exposure, the increase was considerably higher (6.3 ± 0.4 a.u). However, the SNR value was low (7), probably because of the increased noise owing to the greater heterogeneity intensity of the lines. This could be explained by the existence of unreacted PA still on the surface producing variations on the pixel values, due to the different intensities of PA-LSurf and PABF_2_-LSurf.

The results indicated that the detection of BF_3_·O(C_2_H_5_)_2_ vapors is more effective and accurate than the previous detection in solution. Therefore, PA-LSurf surfaces are ideal for the rapid detection of these vapors in industry, providing rapid measurements in 2 min at sort distances of the source (3 cm) without degradation of the surfaces. Moreover, in solution, the response of the surface was also fast (1 min), with a detection limit of 0.5 mM BF_3_·O(C_2_H_5_)_2_.

To verify the formation of the PABF_2_-LSurf and therefore, the presence of the BF_2_–CCMoid adducts, full printed surfaces were further characterized by contact angle measurements and XPS. It is worthy to indicate, that the results agreed well in both approaches, solution and vapor experiments. The contact angle measurements obtained from the PABF_2_-LSurf showed a decrease in the surface polarity induced by the terminal -BF_2_ groups, with an average contact angle of 68° ± 2, Figure S10. The ACA and RCA values were 79^o^ ± 1 and 56° ± 1, respectively. The hysteresis was similar to that observed for the PA-LSurf (23°), displaying therefore the same surface imperfections with still unreacted PACCMoids on the surface, increasing the chemical variability.

**Figure 10:**
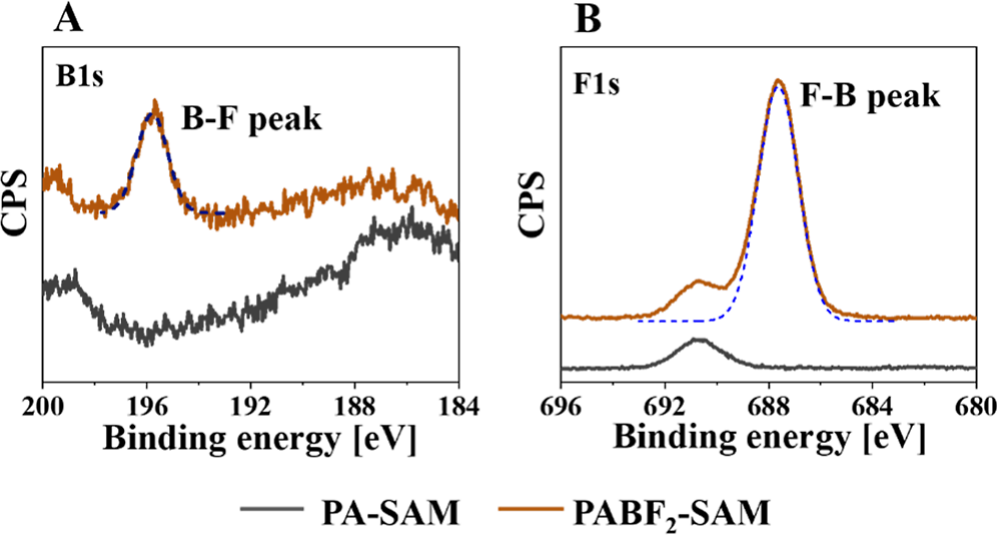
(A) XPS spectra of B1s and (B) F1s of PA-LSurf and PABF2-LSurf by vapor exposure to 10 μL of BF_3_ for 2 min.

As expected, the coordination of PA-LSurfs with -BF_2_ groups involved the observation of a new peak in the XPS B1s spectrum, due to the B–F bond, at 195.7 eV and another in the XPS F1s spectrum at 687.8 eV, due to the F–B bond, Figure 10. In addition, the XPS N1s spectrum showed a decrease at 401.2 eV, agreeing with the loss of additional imidazole groups in the monolayer (Figure S19). This corroborates the instability of the terminal imidazole groups that remained unreacted in PA-LSurf, as they easily degrade with humidity and moisture from the solvents used during the manipulation of the surfaces.

In conclusion, the shift in the fluorescence emission of PABF_2_-LSurf and the observation of the peak assigned to the F–B bond, in the XPS measurements, confirm the reactivity of the PA-functionalized surface to BF_3_. Following these results, studies related to the reversibility of PA coordination with BF_3_ and thus the reusability of these responsive surfaces were carried out.

### Reversibility and Reusability Studies

**Figure 11:**
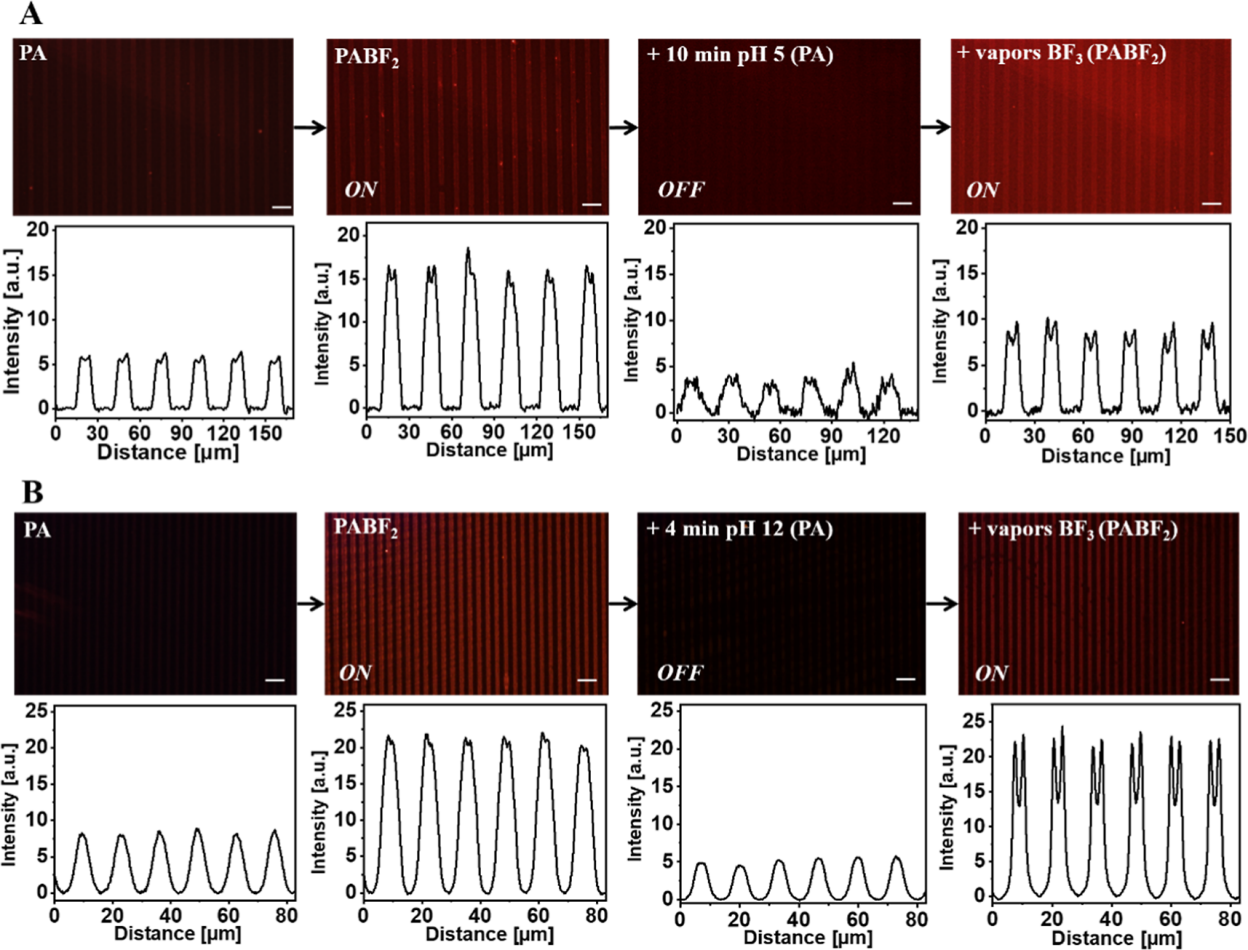
Fluorescence images of PA-LSurf obtained using a green excitation filter. (A) PA-LSurf emission changes upon contact with BF_3_ (ON), after immersion in Milli-Q water for 10 min (OFF), and after reuse for BF_3_ detection (ON). (B) PA-LSurf in contact with BF_3_ (ON), after immersion in basic water for 4 min (OFF), and upon reuse for BF_3_ detection (ON). The images were acquired using a filter with 510 nm ≤ λex ≤550 nm and λem ≥590 nm, ×20 magnification and aperture 8. Scale bar indicates 30 μm.

The reusability of the PABF_2_-LSurf surfaces is an interesting feature, being relevant for their applicability in the design of BF_3_ detectors. Several tests were performed to determine the optimal conditions for removal of the BF_2_ moiety from the surfaces via hydrolysis. The tests using the PABF_2_-LSurf system were based on surface immersion in solutions of different pH values (1, 5, and 12). This way, after 2 min in acidic pH (1–2), the intensity of the lines in the red region decreased but without significant changes; therefore, there was still a high presence of PABF_2_ groups, as it was shown by XPS (F1s and B1s) corroborating the presence of F–B bonds at 687 eV and B–F at 197 eV, respectively (Figure S20). Longer immersion times were unreliable due to damage to the surfaces. Increasing the pH to 5, using Milli-Q water, immersion for up to 10 min was required to significantly decrease the intensity of the patterns. However, lower intensities of the patterns in the visible were also obtained from the reused PA-LSurf, Figure 11A. Moreover, some coordinated PABF_2_ groups still remained, since traces of F–B and B–F peaks were still identified in the XPS spectra (Figure S20). When this PA-LSurf was re-exposed to BF_3_ vapors, the emission was recovered with less intensity and lower contrast between the patterns, suggesting a possible damage of the surface during the process. Nevertheless, when the same process was carried out at pH = 12, upon 4 min of immersion, the intensity of the emission patterns in the NIR decreased significantly and traces of −BF_2_ were only detected, finding a small band in the F1s XPS spectrum, indicating almost complete recovery of the surface, Figures 11B and S20. The further exposition of this substrate (PA-LSurf) to the BF_3_ vapors, showed intensity patterns in the NIR was almost identical to the initial one proving the reversibility and reusability of the PA-LSurf under this conditions, hence, confirming the possibility of performing (ON)–(OFF)–(ON) cycles of the PA-LSurf with BF_3_.

## Conclusions

A fluorescent CCMoid (PA) has been designed to be efficiently immobilized on functionalized surfaces (PA-LSurf) by the use of reactive μCP. First, we show the process of optimizing different parameters, including the printing procedure and the need for humidity control, for the successful generation of fluorescent and active PA-LSurfs. The PA-LSurf proved to be a robust system with high stability that presented PA patterns with emissions in the green region. The shift of the PA emission toward NIR regions upon formation of the −BF_2_ adduct allowed the development of a methodology for the detection of BF_3_ in solution and gas phase. Notably, PA-LSurf provides a fast and easy-to-use system to detect BF_3_, 1 min in solution and 2 min with vapors, compared with other similar systems (based on a change in the optical properties of the material) that require longer times. Moreover, PA-LSurf offers a novel tailor-made system for the direct detection of BF_3_, instead of degraded byproducts in the environment, that to our knowledge, mainly other systems in the literature use. Reversibility and reuse of the surfaces have been demonstrated by immersion in a basic solution performing on–off–on cycles with BF_3_. Finally, PA-LSurf holds promise for further application in portable chemosensors and its coordination to different metal ions is currently under development using, as it is shown here, the emission capacity of these CCMoid-based responsive surfaces.

## Supporting Information Available

Additional experimental details, materials, and methods. Characterization of the compounds including NMR spectra, MALDI-TOF spectra, FTIR-ATR spectra and crystallographic details of PE CCMoid. Printing optimization experiments. Fluorescent microscopy images of control experiments and details of the fluorescence quantification in digital imaging details (PDF)
Crystallographic data of PE CCMoid (CIF) (CIF)
Check CIF (PDF)

## Supplementary Material

am4c19421_si_001.pdf

am4c19421_si_002.cif

## Abbreviations

CCMoid: curcuminoid
CCM: curcumin
PDMS: polydimethylsiloxane
μCP: microcontact printing;
PA: pyrene based CCMoid
PA-LSurf: PA micropatterned surfaces
PABF_2_-LSurf: PA-BF_2_ adduct-based surfaces
SNR: signal-to-noise-ratio
PCA: 1-pyrenecarboxaldehyde
PE CCMoid: PABF_2_ pyrene ester based CCMoid
NMR: nuclear magnetic resonance
FTIR-ATR: Fourier transform infrared-attenuated total reflectance spectroscopy
MALDI-TOF: matrix-assisted laser desorption/ionization
XPS: X-ray photoelectron spectroscopy
TPDEA: N-[3-(trimethoxysilyl)propyl] ethylenediamine]
NIR: near-infrared
THF: tetrahydrofuran
DCM: dichloromethane.
