# Supplementary material for: Curcuminoid-Based Responsive Surfaces for Fluorescent BF3 Detection, a Fast and Reversible Approach
Source: ACS Appl Mater Interfaces. 2025 Mar 25;17(13):20383–93. doi: 10.1021/acsami.4c19421 (PMC11969430; doi:10.1021/acsami.4c19421)
Supplement: Supplementary file 1 — am4c19421_si_001.pdf [file am4c19421_si_001.pdf]

Supporting Information

Curcuminoid-based responsive surfaces for fluorescent BF_3 detection, a fast and reversible approach

Raquel Gimeno-Muñoz[a], Raúl Díaz-Torres[a], Silvia Gómez-Coca[b], Olivier Roubeau[c], José Manuel Díaz-Cruz[d] , Núria Aliaga-Alcalde[a,e*], Arántzazu González-Campo[a*]

[a]Institut de Ciència de Materials de Barcelona (ICMAB-CSIC), Campus de la Universitat Autonoma de Barcelona, 08193, Barcelona, Spain

[b]Departament de Química Inorgànica and Institut de Recerca de Química Teòrica i Computacional, Universitat de Barcelona (UB), Diagonal 645, 08028, Barcelona, Spain.

[c]Instituto de Ciencia de Materiales de Aragón (ICMA), CSIC and Universidad de Zaragoza, Plaza San Francisco s/n, 50009, Zaragoza, Spain.

[d]Departament d'Enginyeria Química i Química Analítica, Universitat de Barcelona (UB), Diagonal 645, 08028, Barcelona, Spain

[e]ICREA (Institució Catalana de Recerca i Estudis Avançats), Passeig Lluís Companys 23, 08010, Barcelona, Spain.

[*]*Corresponding authors*

E mail address: agonzalez@icmab.es

Contents

1. Materials and instruments S-3

2. Synthesis procedures S-5

3. Preparation of PDMS stamps and IM-SAMs S-7

4. Results of the synthesis of PA and PA-BF_2 S-8

5. Characterization of ACAC, PA and $PABF_2$ S-9

6. X-ray crystallography data for PE S-12

7. Contact angle measurements S-14

8. Control studies for the preparation of NH_2-SAM and IM-SAM S-15

9. XPS spectra of IM-SAM and PAL-Surf S-16

10. PA ink concentration studies S-17

11. Printing time studies S-18

12. Stability of the PA-LSurf S-19

13. Blank test: Immersion of PA-LSurf in dry DCM S-19

14. Surface Fluorescence Quantification in Digital Imaging S-20

15. Data analysis of the increase in emission intensity of PA-LSurf when reacting with BF_3 in solution S-22

16. Blank test: Exposure of PA-LSurf to diethyl ether vapors S-23

17. Data analysis of the increase in emission intensity of PA-LSurf when reacting with BF_3 vapors S-23

18. XPS measurements of $PABF_2$-LSurf S-24

19. XPS measurements of the reversibility studies S-25

20. References S-26

1. Materials and Instruments

N-butylamine 99%, tributyl borate 99%, methyl 4-acetyl-5-oxohexanoate, 1-pyrenecarboxaldehyde, hydrochloric acid 37%, rhodamine B isothiocyanate (RBITC), 6-aminofluorescein (6AF), 1,1-carbonyldiimidazole (CDI), N-[3-(trimethoxysilyl)propyl] ethylenediamine] 97% (TPEDA), and boron trifluoride diethyl etherate ($BF_3 \cdot O(C_2H_5)_2$) from Sigma-Aldrich. Lithium hydroxide (LiOH), magnesium sulfate ($MgSO_4$) and boron oxide 99% (B_2O_3) were purchased from ABCR. Dimethylformamide (DMF) and dimethyl sulfoxide-d_6 (DMSO-d_6) by Romil. Absolute ethanol (HPLC Analized) by J. K. Baker. ethyl acetate (EtOAc), diethyl ether and sulphuric acid (96%) (H_2SO_4) from Carlo Erba. Hydrogen peroxide (H_2O_2), dry tetrahydrofuran (THF) (99%) dry and dichloromethane (DCM) (99.9%) by Scharlab. Water Ultrapure AGR WATR-001-10K (18 MΩ) from Aldrich.

Poly(dimethylsiloxane) (PDMS) with sylgard 184 from Dow Corning, glass surfaces (24 x 24 coverslip) from Menzel-Glaser and silicon wafers doped with p-Boron were purchased from University Wafer P.

Solution NMR measurements were recorded on a Bruker Avance DPX 360 MHz spectrometer (8.4 T).

Contact angle measurements were performed with Drop Shape Analyzer DSA 100 from KRÜSS. Milli-Q water droplets were deposited on functionalized surfaces. The DSA 100 uses a computer-controlled automatic system which allowed the following parameters to be set: drop volume of 2 μL and deposition rate of 900 μL / min.

Fluorescence and optical images were obtained using an Olympus RXSITRF with a USH-1030L mercury lamp and a 4-CMAD3 camera to acquire the images.

FTIR-ATR-spectra were collected on a JASCO 4700LE FT-IR spectrometer in the range 400-4000 cm^{-1}.

The EA measurements were conducted using a Thermo Carlo Erba Flash 2000 instrument.

PDMS stamps were tested for oxidation using ozone and oxygen plasma, using the Zepto M2 Plasma Cleaner from Diener Electronics.

The equipment used for characterizing CCMoids was a Bruker ultrafleXtreme MALDI-TOF/TOF mass spectrometer operating at a laser power of 50-70%. The matrix used was *trans*-2-[3-(4-tert-Butylphenyl)-2-methyl-2-propenylidene] malononitrile with a molecular weight of 250.34 g/mol.

Fluorescence measurements in solution and solid state were performed using a Varian fluorescence spectrophotometer (Cary Eclipse). It consists of two slits (excitation and emission) with a double monochromator and a continuously emitting xenon light source between 190 and 900 nm. This includes a range of fixed-width slits and selectable filters.

For the fluorescence measurement of PABF$_2$ in solid state it was required to use the Fluorimetre NanologTM Horib Jobin Yvon IHR320 (detects fluorescence in the near-IR from 800 to 1700 nm) and in addition an integrating sphere was used to measure the quantum yield of PA.

UV-Vis spectra of the compounds of interest were collected using a Jasco V-780 UV-visible/NIR spectrophotometer. This spectrophotometer has a monochromator with auto-switching gratings and detectors for the UV-Visible and NIR regions. The gratings and detectors switch within a user-selected range of 800-900 nm.

XPS was performed using an SPECS Phoibos 150 hemispherical energy analyzer. It was operated with a 3.5 mm beam at 1486.6 eV and 300 W. Data were collected from three areas of PA and PABF$_2$ monolayers on silicon oxide surfaces. An energy of 1 eV was used for the overall scan and high-resolution scans (0.5 eV) in the regions of interest over five cycles.

Confocal microscopy was used to study the stability of a PA-LSURF sample on a glass surface via emission extinction studies Both images and spectra were obtained with a Leica Confocal TCS SP5 (Leica Microsystems GmbH) using a lateral resolution of 200 nm and a 405 nm UV diode laser.

Fluorescence microscopy images were obtained using an Olympus RXSITRF with a USH-1030L mercury lamp and a 4-CMAD3 camera. The filters used were: an excitation filter in the blue region (450 nm $<\lambda ex<$ 480 nm) with emission in the green region $\lambda em \geq$ 515 nm and an excitation filter in the green region (510 nm $\leq \lambda ex \leq$ 550 nm) with emission in the red region $\lambda em \geq$ 590 nm.

2. Synthesis procedures

Synthesis of PE: 301 mg (1.62 mmol) of methyl 4-acetyl-5-oxohexanoate and 120 mg (1.71 mmol) of B_2O_3 were dissolved in 8 mL of ethyl acetate. The reaction mixture was stirred at 60 °C for 1 h. Then, 1.40 mL (5.18 mmol) tributyl borate was added, and the mixture was stirred for 10 min. A second solution was prepared using 500 mg (2.17 mmol) of pyrenecarboxaldehyde in 10 mL of ethyl acetate, which was added to the reaction mixture and stirred for 2 h at the same temperature. The mixture was cooled at room temperature and 0.2 mL (2.02 mmol) of n-butylamine, prepared in 1 mL of ethyl acetate, was added gradually. The mixture was stirred at room temperature for 2 days. The resulting reddish precipitate was filtered off and washed with cold methanol. Finally, the solid was mixed with an acidic aqueous solution (1% HCl), sonicated for 5 min, and then stirred overnight. It was then filtered, washed with cold methanol, and dried with diethyl ether (677 mg, 75% yield). ^{1}H-NMR (DMSO-d$_6$, 360 MHz, ppm): δ 18.10 (s, 1H), 8.93 (m, 2H), 8.69 (m, 2H), 8.40 (m, 6H), 8.30 (m, 6H), 7.79 (m, 3H), 7.44 (m, 1H), 3.58 (s, 2H),

Synthesis of 4-acetyl-5-oxohexanoic acid: 944 mg (5.07 mmol) of methyl 4-acetyl-5-oxohexanoate (1) was dissolved in 10 mL of THF. To this solution 209 mg (8.70 mmol) of LiOH in 10 mL of distilled H_2O was added. The mixture was stirred overnight at room temperature. After this time, THF was removed under vacuum, and 20 mL of 10 % HCl was added and stirred overnight at room temperature. The final compound was extracted with DCM (3 x 20 mL) and dried with $MgSO_4$. After filtration and removal of solvents, a yellow oil of 4-acetyl-5-oxohexanoic acid was obtained, with a Rf = 0.1 in TLCs usign a mixture of hexane: ethyl acetate (1:1) as mobile phase (438 mg, 50 %, yield). ^{1}H-NMR (DMSO-d$_6$, 360 MHz, ppm): δ 16.83 (s, 1H), 12.08 (s, 1H), 2.20 (t, ^3J = 7.3 Hz, 2H), 2.17 (s, 3H), 2.07 (s, 3H), 1.66 (m, ^3J = 7.4 Hz, 2H).

Synthesis of PA: 438 mg (2.37 mmol) of 4-acetyl-5-oxohexanoic acid was mixed with 150 mg (2.14 mmol) of B_2O_3 in 10 mL of ethyl acetate at 60 °C for 1 h, having as a result a white solution. Then, a solution of 2.4 mL (8.89 mmol) of tributyl borate in 1 mL ethyl acetate was added, and the mixture became slightly transparent approximately 10 min later. Then, 797 mg (3.46 mmol) of 1-pyrenocarboxaldehyde in 20 mL of ethyl

acetate were added and the mixture was stirred at 60 °C for 2 h. Then, the solution was cooled to room temperature and 0.2 mL (2.02 mmol) of n-butylamine in 1 mL of ethyl acetate was added dropwise. The reaction mixture was stirred at room temperature for 2 days. Afterward, a boron-CCMoid system was obtained as a reddish-black precipitate, filtered off, dried by mixing with diethyl ether, and washed with cold methanol. Deprotection of the keto-enol moiety from the boron species was carried out by adding the solid to 25 mL of 10 % HCl solution and heating the mixture at 40 °C for 1 h. Then, 80 mL of Milli-Q water was added and the mixture was stirred at room temperature for one day. The reddish-black solid was filtered and recrystallized with hot methanol. For that, the solid was washed several times with methanol, afterwards the mixtures were centrifuged, the solution was removed, and the resulting solid was dried with diethyl ether (665 mg, 66 % yield). FTIR-ATR (cm^{-1}): 3040, 1719, 1590, 1430, 1308, 1227, 837. ^{1}H NMR (DMSO-d_6, 360 MHz, ppm): δ 18.09 (s, 1H), 12.28 (s, 1H), 8.93 (dd, ^{3}J = 15.4 Hz, 2H), 8.73 (dd, ^{3}J= 8.9, 2H), 8.65 (t, ^{3}J= 8.9 Hz, 2H), 8.38 (m, 6H), 8.33-8.18 (m, 6H), 8.13 (dt, ^{3}J= 15.8, 7.8 Hz, 3H), 7.76 (d, ^{3}J= 15.3 Hz, 1H), 7.44 (d, ^{3}J= 15.9 Hz, 1H), 2.61 (t, ^{3}J= 7.3 Hz, 2H), 2.26 (t, J= 7.2 Hz, 2H). ^{13}C NMR (DMSO-d_6, 100 MHz, ppm): δ 196.3, 183.4, 174.4, 174.4, 140.3, 137.7-122.9, 112.1, 59.9, 32.0, 24.2. Elemental analysis (%) calculated for $C_{42}H_{28}O_4{\cdot}0.6$ MeOH: C 83.54, H 4.73, O 10.73; Found: C 83.01, H 5.32, O 11.67. MS MALDI-TOF (m/z): calc. for $C_{42}H_{28}O_4$[M] 596.20; found at $[M-1]^-$ 595.75.

Synthesis of $PABF_2$: 50 mg (0.08 mmol) of PA, 2 mL of dry DCM and 68 µL (0.24 mmol) of $BF_3{\cdot}O(C_2H_5)_2$ were added in a microwave vessel. The mixture was heated by the microwave with stirring at 60 °C for 15 min. This way, a violet precipitate was obtained, filtered and washed with diethyl ether (44 mg, 82 %, yield). FTIR-ATR (cm^{-1}): 3307, 3040, 1709, 1569, 1463, 1308, 1227, 1029, 837. ^{1}H NMR (DMSO-d_6, 360 MHz, ppm): δ 12.4 (s, 1H); 9.18 (d, ^{3}J= 14.8 Hz, 1H); 8,89 (d, ^{3}J= 9.1 Hz, 1H); 8,70 (d, ^{3}J= 7.9, 1H); 8.50-8.10 (m, 9H); 7.91 (d, ^{3}J= 13.8 Hz, 1H); 2.69 (t, 2H); 2.31 (t, 2H). ^{19}F NMR (DMSO-d_6, 250 MHz, ppm): δ 138.3 (s). Elemental analysis (%) calculated for $C_{42}H_{27}BF_2O_4{\cdot}0.35\ CH_2Cl_2$: C 78.27, H 4.22; Found: C 75.23, H 4.38. MS MALDI-TOF (m/z): calc. for $C_{42}H_{27}BF_2O_4$ [M] 644.44; found at $[M-1]^-$ 643.76.

3. Preparation of PDMS stamps and IM-SAMs

3.1 Preparation of PDMS stamps

Stamps were prepared with a mixture of 70 g of poly(dimethylsiloxane) (PDMS) and 7 g of the curing agent (SYLGARD 184 from Dow Corning). The mixture was mixed for 10 minutes and cast over a silicon master (with and without features). Then, air bubbles were removed under vacuum for 1 h and then the mixture was cured for 16 h at 60 °C. The stamps were peeled off the master and cut. They were then oxidized with oxygen plasma for 1 min and stored in Milli-Q water. Before use they were dried with a nitrogen stream.

3.2 Monolayer preparation (IM-SAM)

Microscope glass surfaces were oxidized by immersion in piranha (H_2SO_4 and H_2O_2 in a 3:1 v/v) for 40 min and rinsed with Milli-Q water, drying at last with a nitrogen stream. (Caution: piranha solution should be prepared and handled carefully). In the case of the Si (100) wafers, they were activated with piranha solution for 45 min and after, with basic solution for 30 min (MilliQ-H_2O: H_2O_2: NH_4OH in a 5:1:1 ratio), rinsed with Milli-Q water and finally drying with a nitrogen stream.

For the NH_2-SAMs preparation, the surfaces were placed in a thermostatic vacuum dryer with 0.15 mL of TPEDA, continually pumped for 2 min. After overnight incubation in thermostatic vacuum dryer at 70 °C, the surfaces were cleaned with absolute ethanol, to remove the excess of silanes, immersing the surface in dry DCM and dried with a nitrogen stream.

For the preparation of the IM-SAM, the NH_2-terminated substrates were immersed in a saturated solution of CDI in dry THF for 4 h under argon and covered from light [1]. It is crucial to control the humidity in this step by the fast degradation of the CDI with water. After, the surfaces were rinsed with dry THF and dried with a nitrogen stream.

4. Results of the synthesis of PA and PA-BF_2

The synthesis of PA T-Shape CCMoid started with certain modifications to the Pabon method (Scheme S1). Two synthetic routes were tested for this purpose. The first one (Route A) involved protection of the keto-enol moiety of methyl 4-acetyl-5-oxohexanoate with boron oxide (B_2O_3). This acts as a nucleophile, reacting with the keto-enol group and generating electronic delocalization throughout the system. In this way, Knoevenagel condensation is avoided. Next, aldol condensation at the terminal carbons was promoted after reaction with 1-pyrenecarboxaldehyde (PCA) and N-butylamine (n-$BuNH_2$) as the reaction catalyst. Deprotection was then carried out to obtain PE CCMoid (which had a central ester termination) in 75 % yield.

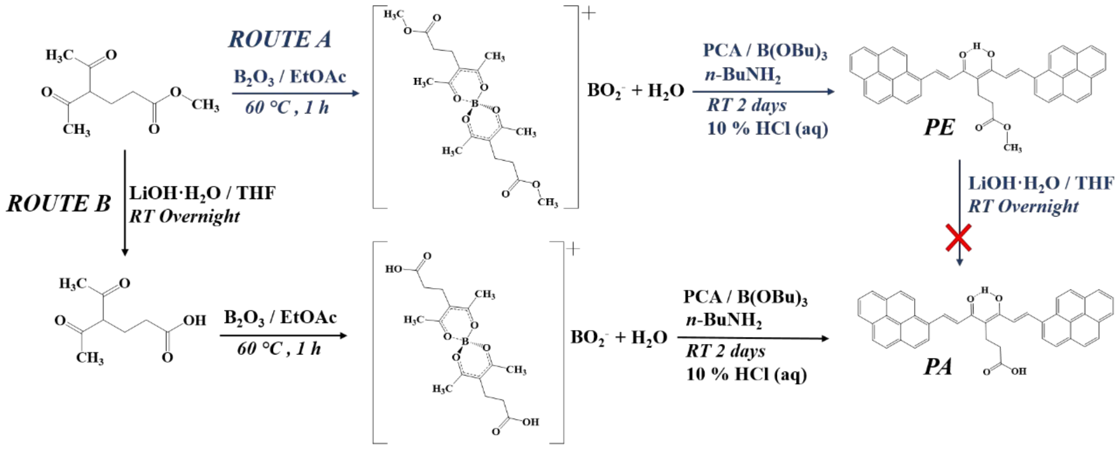

Scheme S1. *Synthetic routes for the synthesis of PA CCMoid.*

Owing to the solubility problems that hindered the correct hydrolysis of the ester group to carboxylic acid, route B was proposed. Route B was mainly based on the hydrolysis of methyl 4-acetyl-5-oxohexanoic acid by its analogous ester. Once the acid moiety was synthesized, the same synthesis as described for Route A was performed, and PA CCMoid was successfully obtained with a yield of 66%.

With the objective to study the fluorescent properties of PA upon complexation with BF_3, the corresponding boron difluoride complex CCMoid (PABF$_2$) was also prepared (Scheme S2). For this purpose, PA was mixed with $BF_3{\cdot}O(C_2H_5)_2$ in DCM. The reaction mixture was exposed to microwave energy and a PABF$_2$ was obtained in high yield.

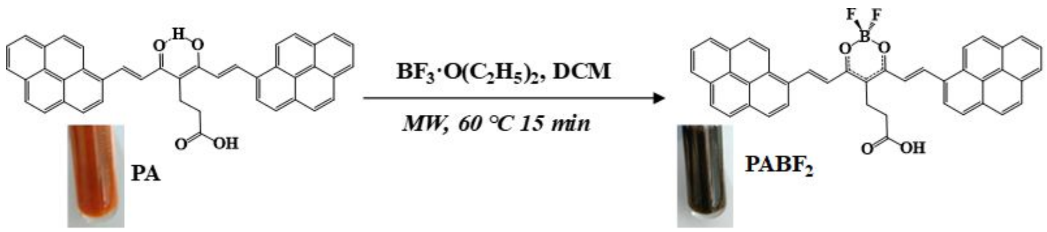

Scheme S2. *Synthetic route for the coordination of PA with BF_3 (PA-BF_2) by MW energy.*

5. Characterization of ACAC, PA and PABF$_2$

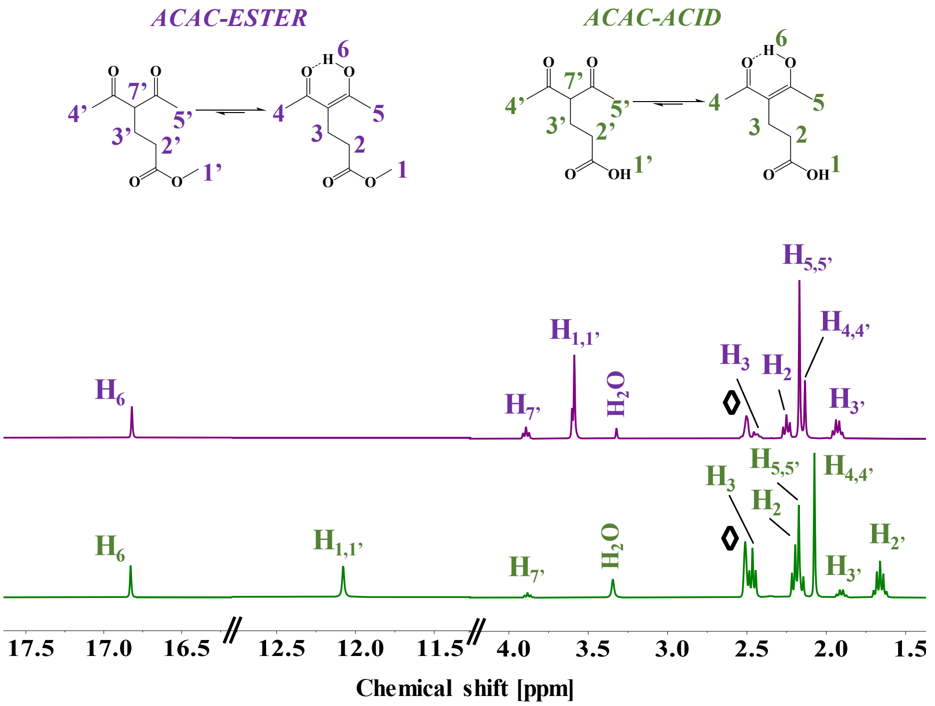

Figure S1. *^{1}H NMR spectra and signals assigned to ACAC-ester and ACAC-acid (◊ DMSO-d$_6$).*

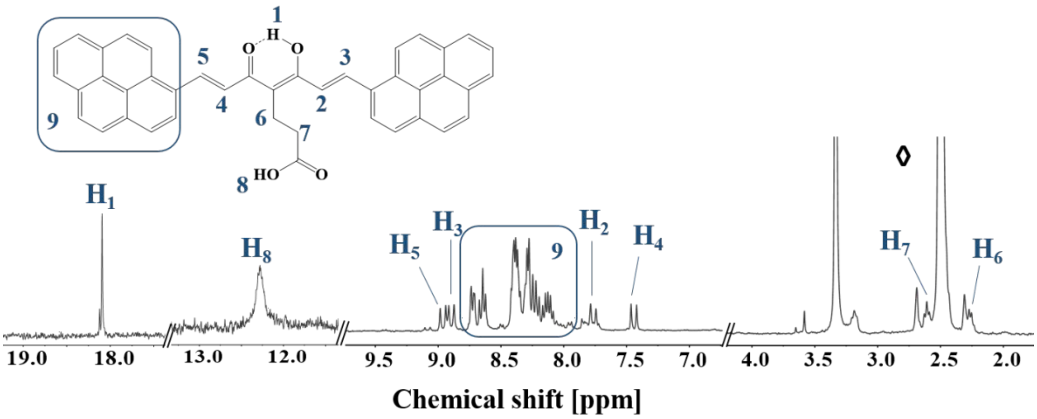

Figure S2. *^{1}H NMR spectrum and assigned signals of PA (◊ DMSO-d$_6$).*

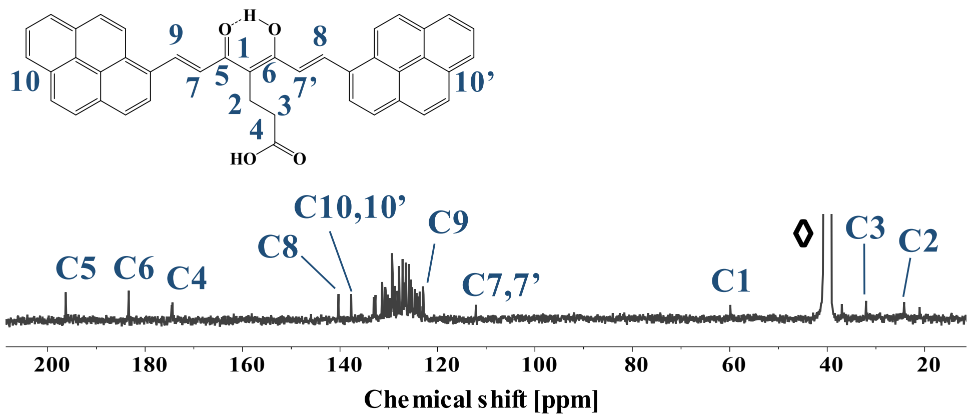

Figure S3. *^{13}C NMR spectrum and signals assigned to PA (◊ DMSO-d$_6$).*

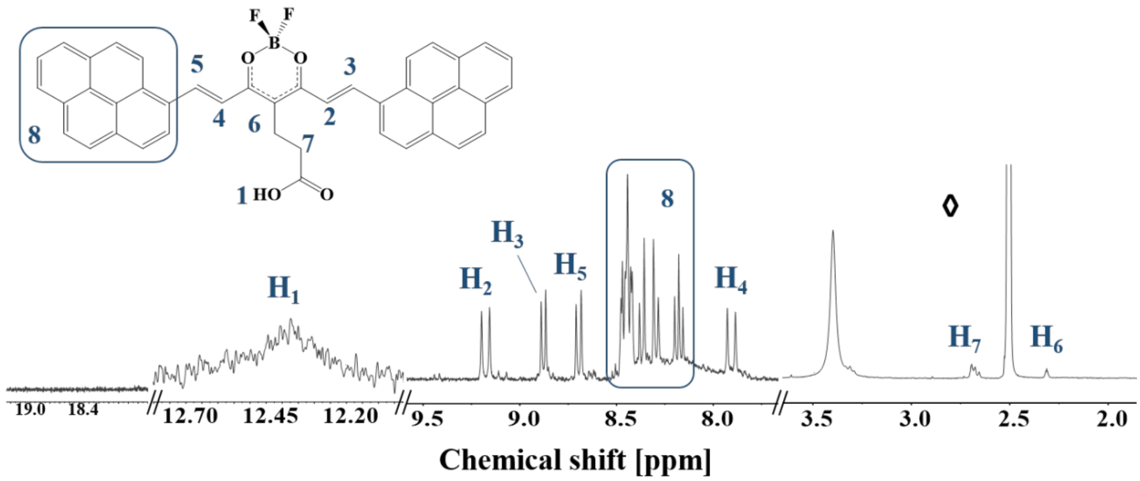

Figure S4. *^{1}H NMR spectrum and assigned signals of PABF$_2$ (◊ DMSO-d$_6$).*

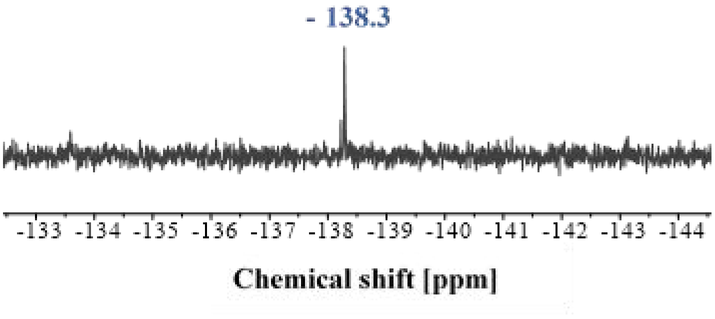

Figure S5. *^{19}F NMR spectrum and assigned signals of PABF$_2$ (DMSO-d$_6$).*

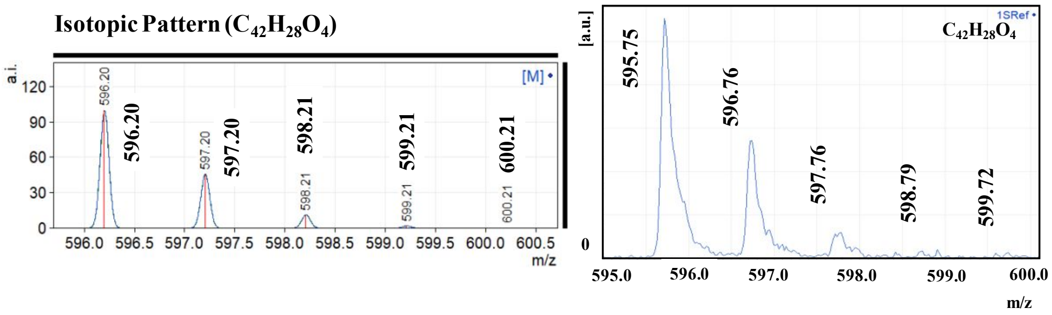

Figure S6. *Isotopic pattern calculated for $C_{42}H_{28}O_4$ (PA CCMoid); and mass spectrum obtained for PA CCMoid.*

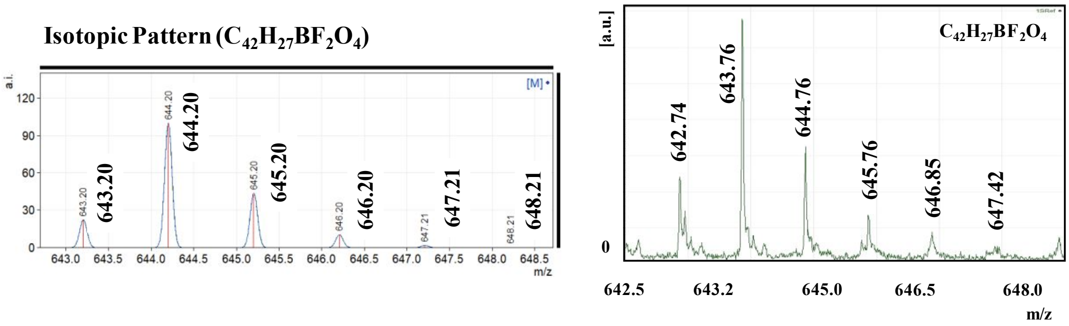

Figure S7. *Isotopic pattern calculated for $C_{42}H_{27}BF_2O_4$ ($PABF_2$); and mass spectrum obtained for $PABF_2$ CCMoid.*

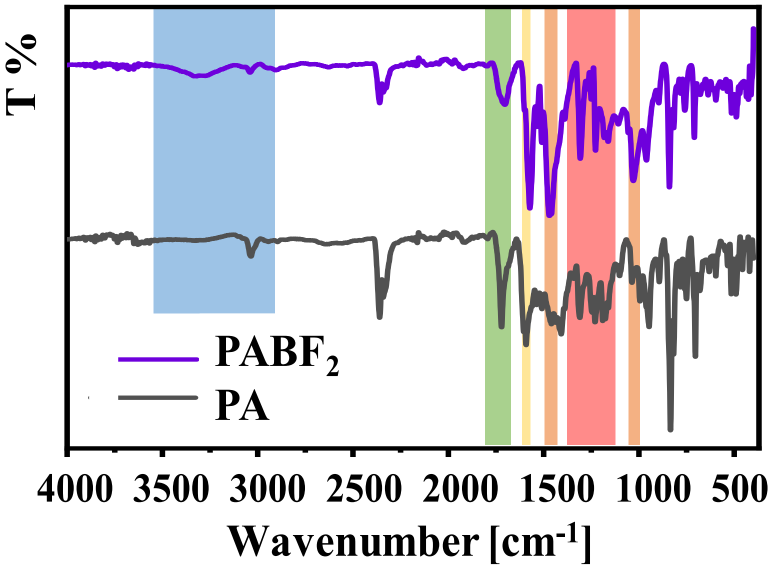

Figure S8. *FTIR-ATR spectra of the PA (black) and $PABF_2$ (purple).*

6. X-ray crystallography of PE

PE crystals were obtained from a solution of CH_2Cl_2. Data for compound **PE** were obtained at 100 K on a 0.09 x 0.05 x 0.03 mm^3 red plate at Beamline 11.3.1 of the Advanced Light Source (Berkeley, USA), on a Bruker D8 diffractometer equipped with a PHOTON 100 detector and using silicon (111) monochromated synchrotron radiation (l = 0.7749 Å). Data reduction and absorption corrections were performed with SAINT and SADABS, respectively [2]. The structure was solved by intrinsic phasing with SHELXT, [3] and refined by full-matrix least-squares on F^2 with SHELXL [4]. No Flack parameter value is reported as it took unrealistic (>1) values with similarly large s.u. This is likely due to the absence of any anomalous diffraction associated with no atom heavier than Si being present in the structure. All details can be found in CCDC 2373749 (**PE**) that contain the supplementary crystallographic data for this paper. These data can be obtained free of charge from The Cambridge Crystallographic Data Center via https://www.ccdc.cam.ac.uk/structures/. Crystallographic and refinement parameters are summarized in Table S1. Details of intramolecular hydrogen bonds are given in Tables S2.

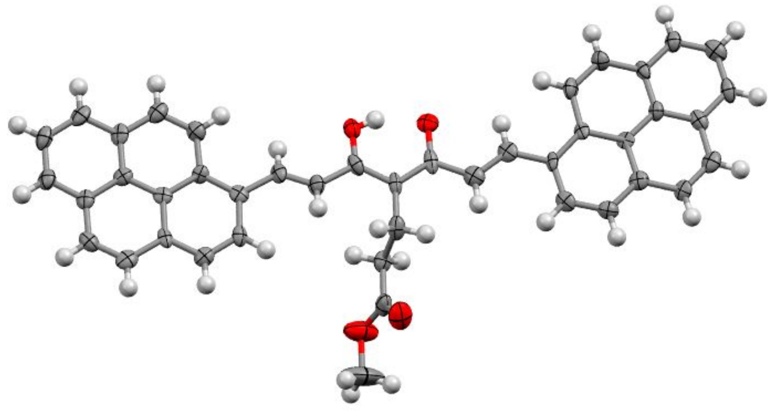

Figure S9. *Crystal structure of PE*

Table S1. *Crystallographic and refinement parameters for the structures of compound* ***PE****.*

	**PE**
Formula	$2(C_{43}H_{30}O_4)$
FW (g mol^{-1})	1221.34
T (K)	100
Wavelength (Å)	0.7749
Crystal system	orthorhombic
Space group	*A*ba2
a (Å)	35.0440(19)
b (Å)	45.145(2)
c (Å)	7.6689(4)
α (°)	90
β (°)	90
γ (°)	90
V ($Å^3$)	12132.8(11)
Z	8
ρ_{calcd} (g cm^{-3})	1.337
μ (mm^{-1})	0.101
Independent reflections (R_{int})	4784 (0.0612)
parameters / restraints	911 / 212
Goodness-of-fit	1.077
Final R1 / wR2 [I>2σ(I)]	0.0474 / 0.1077
Final R1 / wR2 [all data]	0.0666 / 0.1173
largest diff. peak / hole (e $Å^3$)	0.479 / –0.186

Table S2. *Intramolecular hydrogen bonds in the structures of* ***PE****.*

D–H···A	D–H (Å)	H···A (Å)	D···A (Å)	D–H···A (°)
O4–H4···O3	0.80(11)	1.68(11)	2.420(9)	153(12)
O7–H7···O8	0.90(11)	1.56(12)	2.414(9)	159(10)

7. Contact angle measurements

The contact angle measurements of PA-LSurf showed a static contact angle of 81° ± 3, indicating a clear change in surface polarity compared to IM-SAMs (57°), Figure S10. Moreover, the relatively large hysteresis value (22°) obtained from the advancing (ACA, 86° ± 2) and receding (RCA, 64° ± 1) measurements, indicated that monolayer presents variations, and most likely related to the fact that not all the imidazole groups reacted with PA due to steric hindrance.

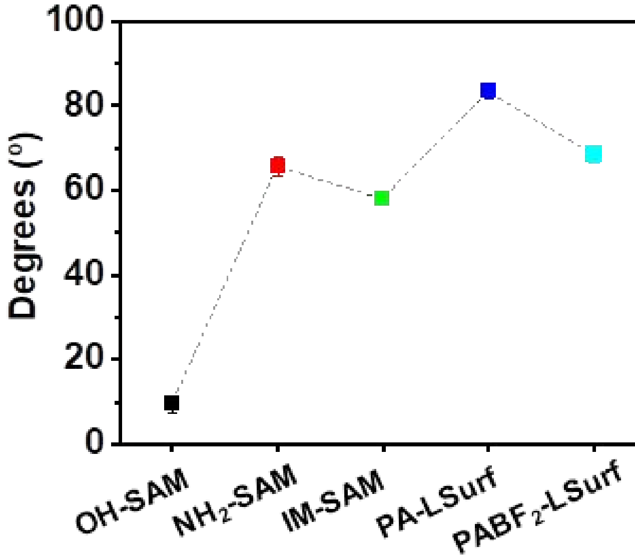

Figure S10. *Evolution of contact angle measurements of full functionalized surfaces for activation, NH_2-SAM, IM-SAM, PA-LSurf and $PABF_2$-LSurf.*

8. Control studies for the preparation of NH_2-SAM and IM-SAM

To monitor the preparation of the IM-SAM monolayer, controls were prepared by printing fluorescent dyes, such as rhodamine B isothiocyanate (RBITC) or 6-aminofluorescein (6AF), onto the NH_2-SAM and IM-SAM monolayers

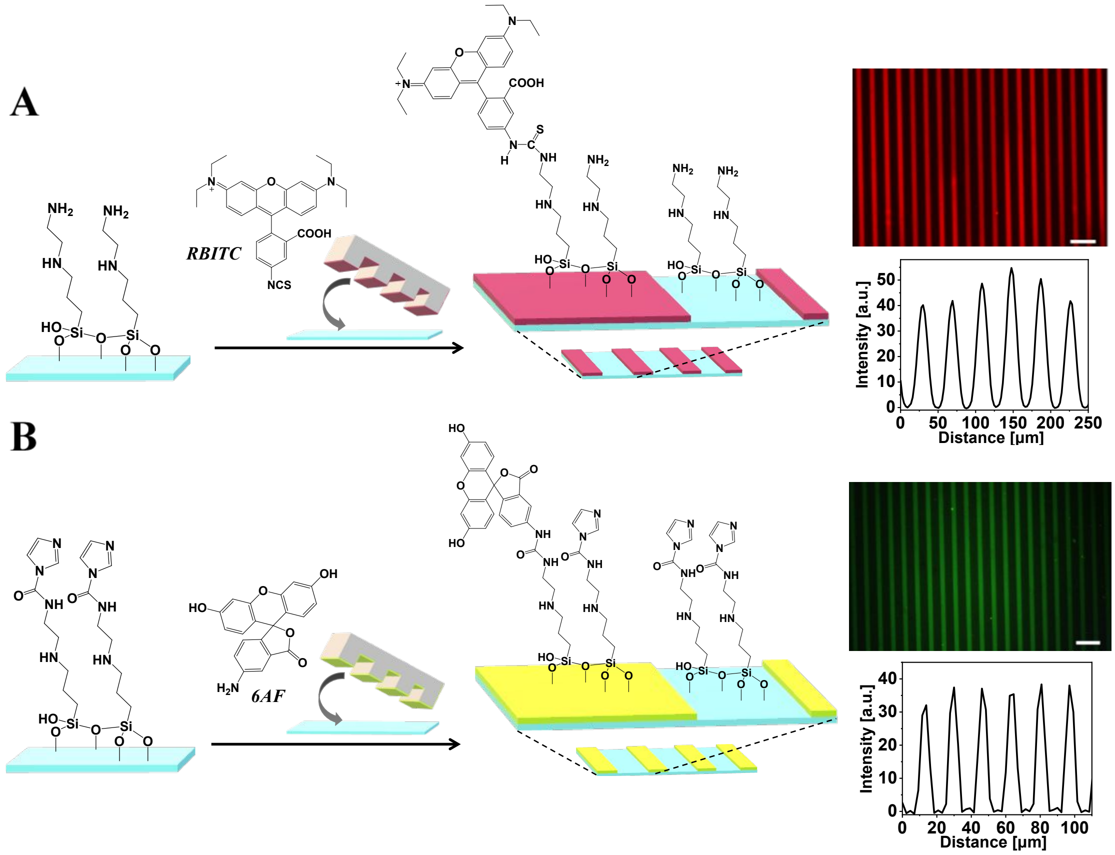

Figure S11. *Fluorescent microscopy images of micropatterns of 5 µm lines of* ***(A)*** *RBITC, using an excitation filter of 510 nm ≤ λex ≤ 550 nm and emission at λem ≥ 590 nm, and* ***(B)*** *6 AF with an excitation filter of 450 nm < λex < 480 nm and emission at λem ≥ 515 nm. The images were acquired at a magnification of x20 and an aperture of 8. Scale bar: 30 microns. The plot profile was studied using ImageJ-FIJI*

9. XPS spectra of IM-SAM and PAL-Surf

When comparing the XPS C1s spectrum of PA-LSurf with that of IM-SAM, an increase in the C=O peaks (286.5 eV) was observed owing to the presence of keto-enol moiety of the attached CCMoid, Figure S12. The peaks at 284.8, 285.9, 287.7, and 291 eV correspond to C-(C,H)/C-Si, C-(N,O), N-C=O/N-C(O)-N, and π–π satellite, respectively. Therefore, the formation of an amide bond was confirmed and with that the covalent anchoring of PA to the IM-SAM; the existence of C-N bonds is related to the presence of amines in the chain of the IM-SAM (unreacted imidazole end groups). The XPS N1s spectrum from the PA-LSurf also maintained two peaks at 401 and 402.4 eV, presenting a lower intensity at 401 eV, agreeing with the loss of terminal imidazole groups when reacting with PA.

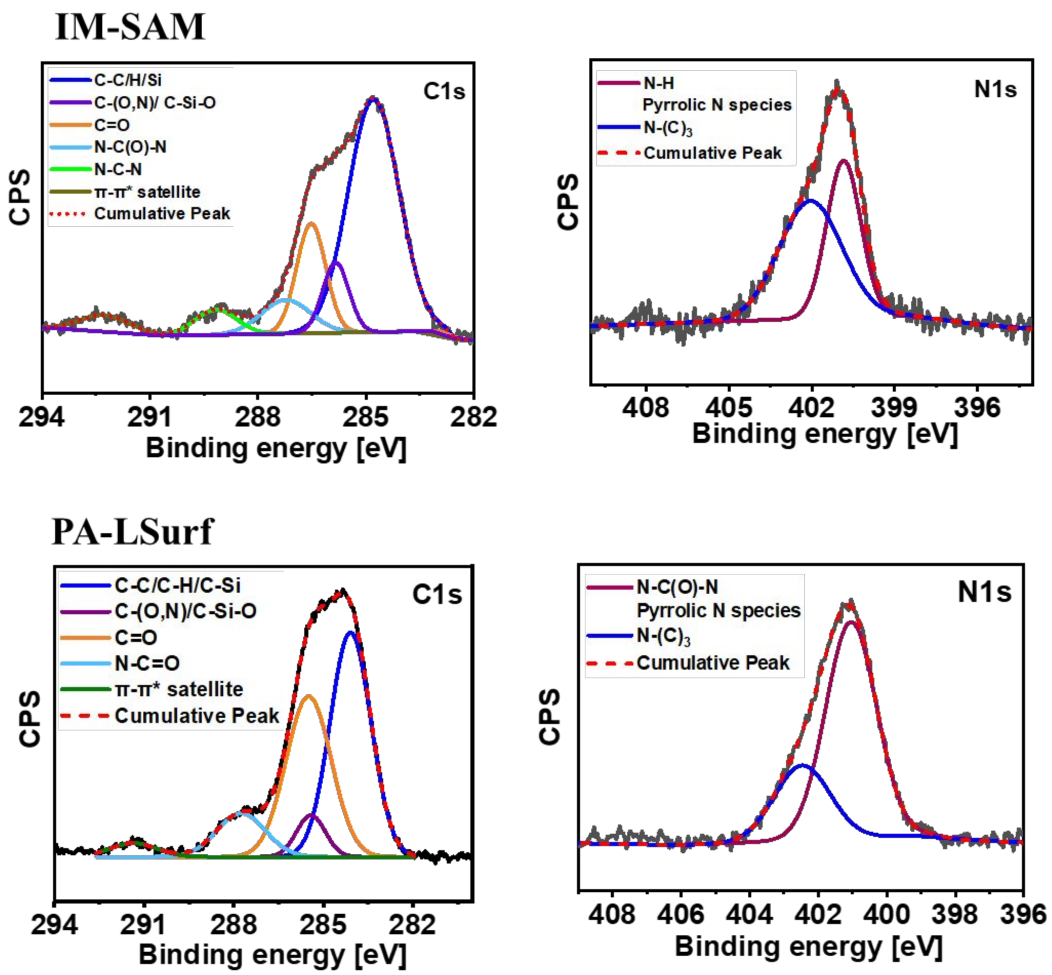

Figure S12. *Deconvolution of the XPS spectra of the IM-SAM, and PA-LSurf (C1s region and N1s region).*

10. PA ink concentration studies

The ink concentrations evaluated were from 10^{-6} M to $2{\cdot}10^{-3}$ M of PA in DMF, shown in Figure S13A. When the lowest concentration of 10^{-6} M was used, no patterns were observed. Detection of the emission of PA started by increasing the concentration to 10^{-5} M, and therefore, the presence of micropatterns was observed. However, the fluorescence intensity values of the patterns at 10^{-5} M and 10^{-4} M were low, indicating that insufficient material was immobilized.

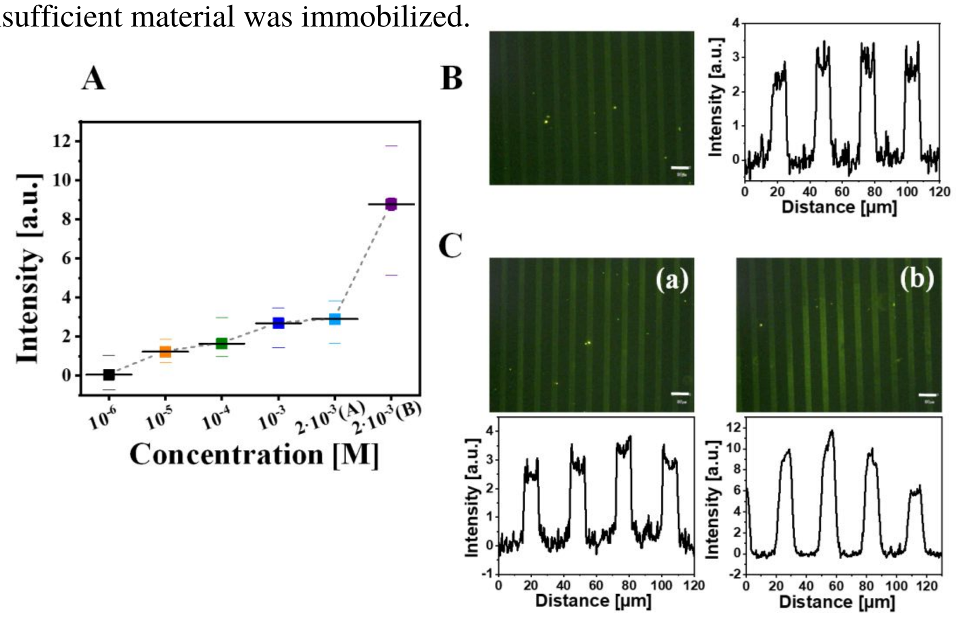

Figure S13. *A) Study of the emission intensity of PA-LSurf at different PA concentrations during incubation of the stamp (inking), graphical representation of the mean emission intensity on the surface for each PA concentration. In addition, the maximum and minimum intensity values acquired for each study are shown. Fluorescence images of PA-LSurf surfaces after microcontact printing and the corresponding plot profile with:* ***B)*** *10^{-3} M PA and* ***C)*** *$2{\cdot}10^{-3}$ M PA, where both images (a-b) show different areas of the same surface. Scale bar: 30 µm*

Using higher concentrations, starting at 10^{-3} M, more homogeneous patterns were obtained, which showed higher emission intensities (Figure S13B). However, using a $2{\cdot}10^{-3}$ M concentration, the pattern was not homogeneous and showed presence of aggregates in some areas (Figure S13C), probably due to the formation of multilayers of PA on the surfaces.

11. Printing time studies

For the printing time optimization study, PA ink solutions of 10^{-3} M and $2\cdot10^{-3}$ M in DMF were used. The surfaces were stored in a vacuum desiccator during the printing to regulate the humidity and prevent degradation of the terminal imidazole groups of the IM SAMs. Patterns were observed after 1 h of printing, with the maximum intensity observed after 3 h for both PA concentrations (Figure S14). Nevertheless, longer printing times maintained a good average intensity by controlling the humidity, resulting in more homogeneous patterns with good contrasts. This way, the PA emission intensities were reproducible, with a mean of 12-15 a.u. intensity values.

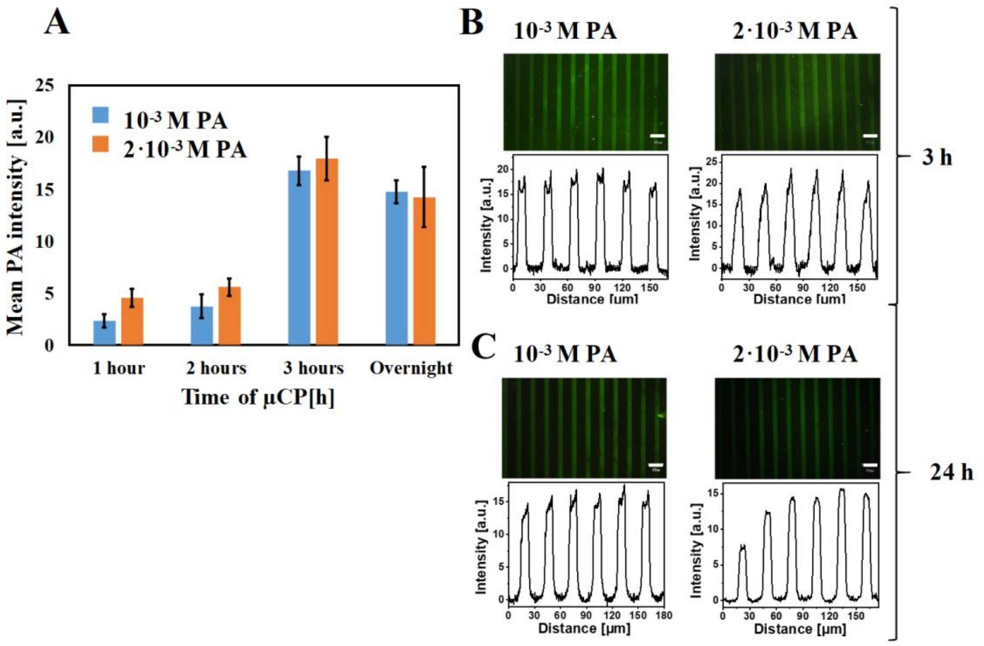

Figure S14: *A) Printing time optimization study, graph of mean intensities over time for stamps incubated with 10^{-3} M PA and $2\cdot10^{-3}$ M PA. Fluorescent microscopy PA-LSurf images, and their corresponding intensity plot profiles ((filter 450 nm < λex < 480 nm; λem ≥ 515 nm) after* ***B)*** *3 h or* ***C)*** *24h of printing. Magnification: x20 and aperture:8, scale bar: 30 µm).*

12. Stability of the PA-LSurf

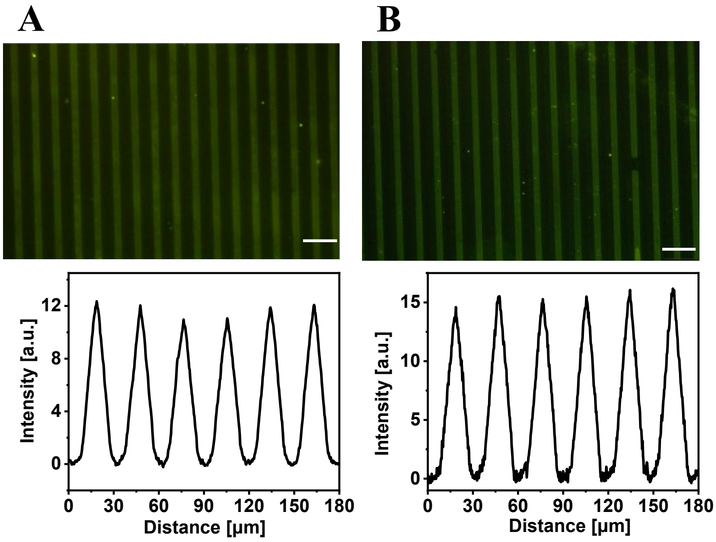

Figure S15. A) *Fluorescent microscopy images of PA-LSurf and* ***B)*** *of the same PA-LSurf surface after two weeks storage under inert conditions. Scale bar: 50 µm.*

13. Blank test: Immersion of PA-LSurf in dry DCM

The PA-LSurf surface was immersed for 1 min in a dry DCM solution, then dried with a stream of nitrogen, and inspected using a fluorescence microscope. To do this, the sample was excited in the blue region, and the images in Figure S16 were acquired using the same acquisition parameters selected for all assays of PA-LSurf.

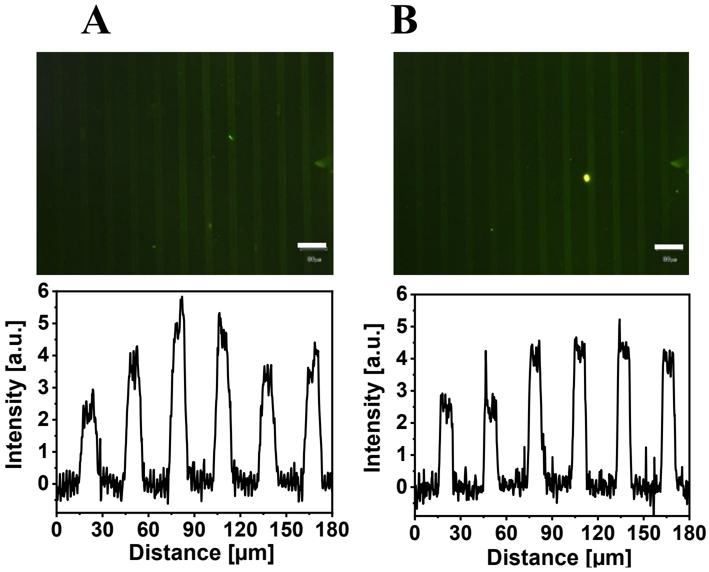

Figure S16. A) *Image obtained from PA-LSurf,* ***B)*** *PA-LSurf surface after 1 min of immersion in dry DCM, and* ***C)*** *plot profile of surface before and after immersion. Scale bar: 30 µm.*

14. PA-LSurf and PABF$_2$-LSurf Fluorescence Quantification in Digital Imaging

All emission analyses of the PA-LSurf system were performed using digital images acquired using a fluorescence microscope. The quantification of the optical intensity required that both, the PA-LSurf preparation methodology and the image acquisition, were always carried out with the same exact parameters, in order to be able to compare the results between different samples. Therefore, all the PA-LSurf surfaces were prepared using the same reagents, days, and conditions. They were inspected with an Olympus RXSITRF microscope using the same excitation filters, a USH-1030L mercury lamp, and a 4-CMAD3 camera. Images were acquired at the same magnification (x20), exposure (8s), and gain (x2) parameters. In this manner, images that could be compared were acquired. Furthermore, they were saved in TIFF format to correctly quantify fluorescence without producing changes in pixel values.

Acquisition parameters were determined to ensure that the sample with the highest intensity did not exhibit signal saturation. With the same parameters, the lowest intensity samples should be observed with minimal noise to avoid the loss of information and allow comparison during optimization studies.

To determine the detection limit, data analysis was performed using the following steps:

Step 1. *An intensity plot profile (intensity vs. distance) was achieved, taking into account a representative surface area of the entire sample, followed by the correction on the plot profile to remove background noise, taking the sample blank (unreacted imidazole terminal area) as zero.*

Step 2. *The average intensities of the lines PA-LSurf (initial intensity,* I_0*) and PABF$_2$-LSurf (final intensity,* I_f*) were calculated from the values of the plot profile. These values were determined for each test at different BF$_3$ concentrations together with the standard deviation of the signals. From this information it was possible to determine the signal-to-noise ratio (SNR) for each surface.*

Eqs. 1 and 2 were used to calculate the average intensity ($\bar{x}$) and the standard deviation (***S***) as it follows:

$$\bar{x} = \frac{\sum_{i=1}^{N} x_i}{N} \quad \textbf{(1)}$$

$$S = \sqrt{\frac{\sum_{i=1}^{N} (x_i - \bar{x})^2}{N-1}} \quad \textbf{(2)}$$

Where x_i represents the different intensity values and N is the number of measurements, which were 88.

Step 3. *Next, the increase in the intensity generated by the reaction of PA with* BF_3*, giving as a result the* $PABF_2$ *systems, was calculated using Eq.3, where* I_f *and* I_0 *represent the final and initial average intensities, respectively. The absolute standard deviation was calculated by applying the error propagation method [5]. Considering that* I_0 *and* I_f *are not correlated, Eq. 4 was applied, where* σ_f *and* σ_0 *are the standard deviations associated with the average of* I_f *and* I_0*, respectively [5][6].*

$$\frac{I_f - I_0}{I_0} \quad \textbf{(3)}$$

$$\sigma = \sqrt{\frac{\sigma_f^2 + \sigma_0^2}{(I_f - I_0)^2} + \frac{\sigma_0^2}{(I_0)^2}} \quad \textbf{(4)}$$

This limitation, comes from the working microscope and the sample and makes the initial intensity high enough to be considered in the quantification of the final intensity of the PA lines after their coordination with the BF_3 groups. In addition, not all PA-LSurf surfaces present the same initial intensity (experimental fluctuations); therefore, to be able to compare samples where different BF_3 concentrations have been used, it is necessary to subtract the initial intensity from the final acquired intensity in all cases.

For this reason, these studies did not exclusively focus on the value of the final intensity acquired, but on the increase in intensity (difference between the final and initial intensity, Figure S17).

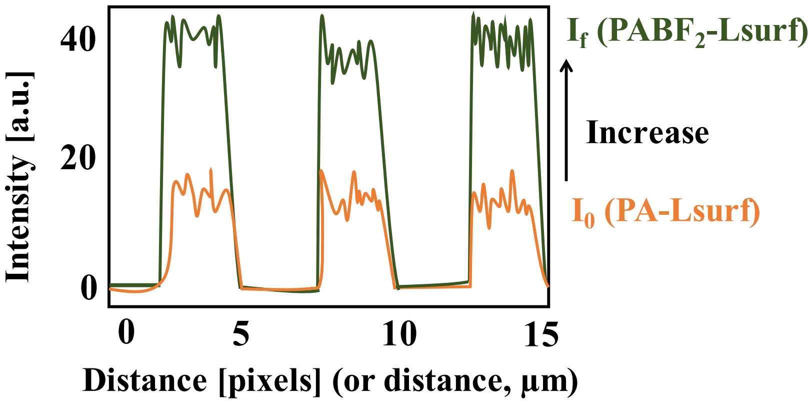

Figure S17. *Representation of the increase in emission intensity (in the red region) of the trace profile obtained from the PA-LSurf surface before and after exposure to BF_3.*

Step 4. *The results obtained in Step 3 can be used to plot the intensity against the BF_3 concentration in absolute values and to obtain a trend line that allows the quantification of BF_3 from the intensity of other PA-LSurf samples that react to BF_3.*

15. Data analysis of the increase in emission intensity of PA-LSurf when reacting with BF_3 in solution (DCM).

Table S3. *Analysis of $PABF_2$-LSurf intensity data from assays in solution (BF_3 in DCM)*

5 µm lines of $PABF_2$	I_F-I_0/I_0	σ	SNR	10 µm lines of $PABF_2$	I_F-I_0/I_0	σ	SNR
$2.5\cdot10^{-4}M$	-	-	-	*$2.5\cdot10^{-4}M$*	2.2	0.3	8
$5\cdot10^{-4}M$	3.2	0.2	11	*$5\cdot10^{-4}M$*	0.4	0.7	6
$7.5\cdot10^{-4}M$	1.4	0.2	17	*$7.5\cdot10^{-4}M$*	1.1	0.4	5
$1\cdot10^{-3}M$	2.1	0.2	13	*$1\cdot10^{-3}M$*	1.1	0.5	5
$1.5\cdot10^{-3}M$	2.0	0.4	5	*$1.5\cdot10^{-3}M$*	1.7	0.3	7
$2\cdot10^{-3}M$	9.4	0.2	13	*$2\cdot10^{-3}M$*	2.8	0.2	11
$2.3\cdot10^{-3}M$	-	-	-	*$2.3\cdot10^{-3}M$*	4.2	0.1	10

16. Blank test: Exposure of PA-LSurf to diethyl ether vapors

The PA-LSurf surface was exposed to 15 µL diethyl ether for 2 min. For this, the same setup used for all tests with $BF_3{\cdot}O(C_2H_5)_2$ was used, which allowed the surface to be 3 cm away from the focus and in a closed system. The tests were conducted at 25 °C. Subsequently, the surfaces ware washed with dry DMC and dried under a nitrogen stream. Finally, the surfaces were observed under a fluorescence microscope. The samples were excited in green region, and images in Figure S18 were acquired using the same acquisition.

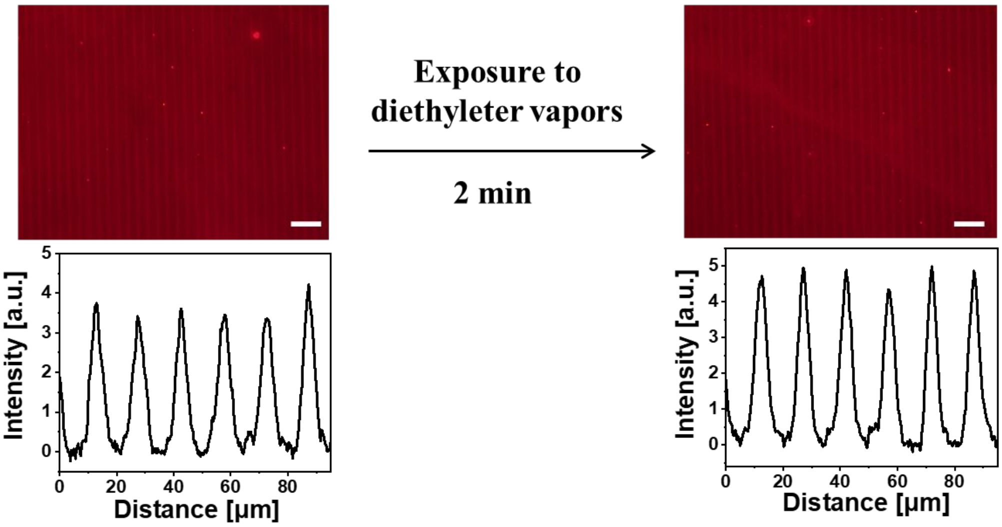

Figure S18. *PA-LSurf fluorescence microscopy images obtained after 2 min of exposure to diethyl ether using. Scale bar: 50 µm.*

17. Data analysis of the increase in emission intensity of PA-LSurf when reacting with BF_3 vapors.

Table S4. *Analysis of $PABF_2$-LSurf intensity data from BF_3 vapors exposure*

1 min	I_F-I_0/I_0	σ	SNR	2 min	I_F-I_0/I_0	σ	SNR
2 µL	1.7	0.3	8	***2 µL***	1.9	0.3	7
4 µL	0.7	0.6	4	***4 µL***	2.3	0.3	6
6 µL	2.6	0.3	7	***6 µL***	2.6	0.4	7
8 µL	0.9	0.5	7	***8 µL***	4.3	0.4	7
10 µL	1.9	0.4	8	***10 µL***	5.4	0.2	18
15 µL	3.3	0.2	13	***15 µL***	6.3	0.4	8

18. XPS measurements of $PABF_2$-LSurf

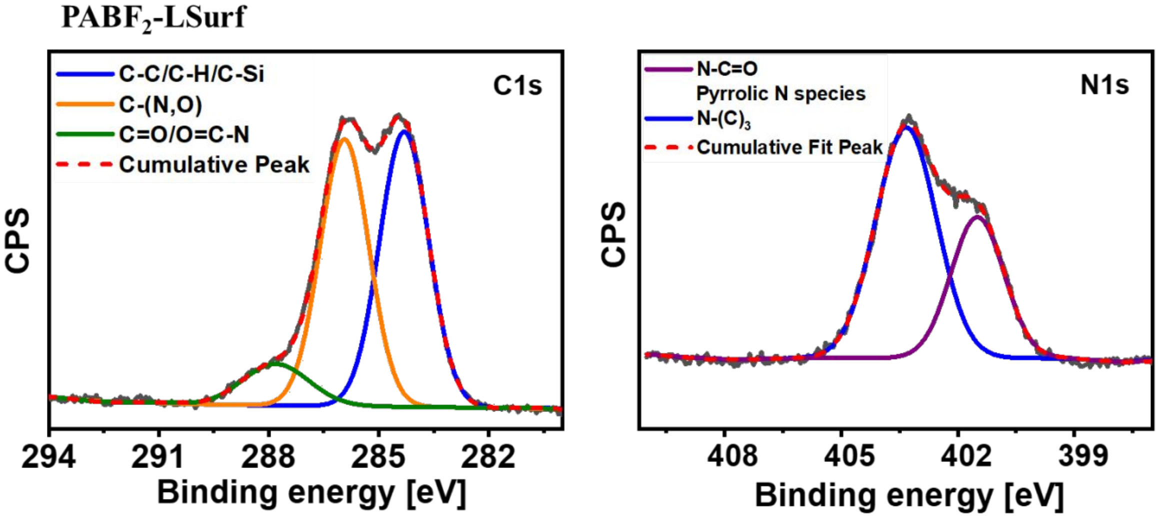

Figure S19. *Deconvolution of the XPS spectrum of the $PABF_2$-LSURF (C1s region and N1s region).*

19. XPS measurements of the reversibility studies

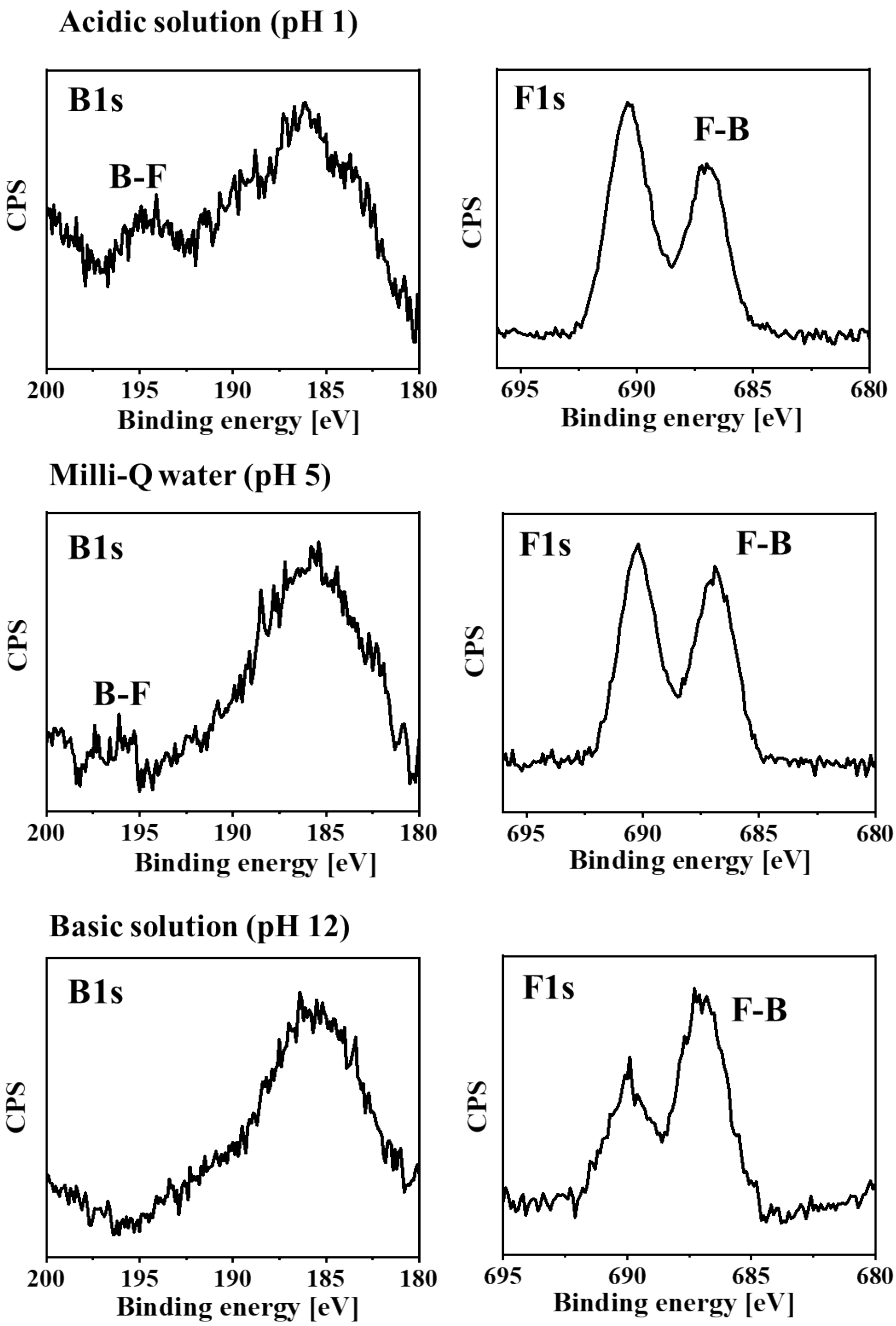

Figure S20. *XPS spectra of B1s and F1s of PABF$_2$-LSurf after immersion in pH= 1, pH 5 and pH 12.*

20. References

[1] S. H. Hsu, D. N. Reinhoudt, J. Huskens, A. H. Velders, Imidazolide monolayers for reactive microcontact printing, *J. Mater. Chem.*, **2008**, *18*, 4959-4963.

[2] G. M. Sheldrick, 2014, *SAINT and SADABS*, Bruker AXS Inc., Madison, Wisconsin, USA.

[3] G. M. Sheldrick, *Acta Cryst. A*, **2015**, *71*, 3-8.

[4] G. M. Sheldrick, *Acta Cryst. C*, **2015**, *71*, 3-8.

[5] Douglas W Cromey, Avoiding twisted pixels: ethical guidelines for the appropriate use and manipulation of scientific digital images. *Science and Engineering Ethics* **2010**, *16*, 639-667.

[6] J. Miller, J.C. Miller, Statistics and chemometrics for analytical chemistry. Pearson education, **2018**.
