# Supplementary material for: Curcuminoid-Based Responsive Surfaces for Fluorescent BF3 Detection, a Fast and Reversible Approach
Source: ACS Appl Mater Interfaces. 2025 Mar 25;17(13):20383–93. doi: 10.1021/acsami.4c19421 (PMC11969430; doi:10.1021/acsami.4c19421)

checkCIF/PLATON report

Structure factors have been supplied for datablock(s) rdt059

THIS REPORT IS FOR GUIDANCE ONLY. IF USED AS PART OF A REVIEW PROCEDURE FOR PUBLICATION, IT SHOULD NOT REPLACE THE EXPERTISE OF AN EXPERIENCED CRYSTALLOGRAPHIC REFEREE.

No syntax errors found. CIF dictionary Interpreting this report

Datablock: rdt059

Bond precision:	C-C = 0.0163 A	Wavelength=0.77490	
Cell:	a=35.0440(19)	b=45.145(2)	c=7.6689(4)
	alpha=90	beta=90	gamma=90
Temperature:	100 K		

	Calculated	Reported
Volume	12132.7(11)	12132.8(11)
Space group	A b a 2	A b a 2
Hall group	A 2 -2ac	A 2 -2ac
Moiety formula	C43 H30 O4	2(C43 H30 O4)
Sum formula	C43 H30 O4	C86 H60 O8
Mr	610.67	1221.34
Dx,g cm-3	1.337	1.337
Z	16	8
Mu (mm-1)	0.101	0.101
F000	5120.0	5120.0
F000'	5122.98	
h,k,lmax	31,40,7	31,40,6
Nref	4815[2671]	4784
Tmin,Tmax	0.994,0.997	0.567,0.745
Tmin'	0.991	

Correction method= # Reported T Limits: Tmin=0.567 Tmax=0.745
AbsCorr = MULTI-SCAN

Data completeness= 1.79/0.99 Theta(max)= 20.718

R(reflections)= 0.0474(3832) wR2(reflections)= 0.1173(4784)

S = 1.077 Npar= 911

The following ALERTS were generated. Each ALERT has the format
test-name_ALERT_alert-type_alert-level.
Click on the hyperlinks for more details of the test.

Alert level A

```
THETM01_ALERT_3_A  The value of sine(theta_max)/wavelength is less than 0.550
            Calculated sin(theta_max)/wavelength =    0.4565
```

Author Response: Crystals were small and poorly diffracting. Even using a synchrotron source significant diffraction with reasonable completeness was only up to ca. 1.05 angstrom. The present data were cut at 1.10 angstrom resolution, as then Rmerge > 30%. This results in poor theta_full and theta_max values, as well as poor data/parameters ratio.

```
PLAT027_ALERT_3_A _diffrn_reflns_theta_full value (too) Low ......      20.62 Degree
```

Author Response: Crystals were small and poorly diffracting. Even using a synchrotron source significant diffraction with reasonable completeness was only up to ca. 1.05 angstrom. The present data were cut at 1.10 angstrom resolution, as then Rmerge > 30%. This results in poor theta_full and theta_max values, as well as poor data/parameters ratio.

```
PLAT089_ALERT_3_A Poor Data / Parameter Ratio (Zmax < 18) ........       2.91 Note
```

Author Response: Crystals were small and poorly diffracting. Even using a synchrotron source significant diffraction with reasonable completeness was only up to ca. 1.05 angstrom. The present data were cut at 1.10 angstrom resolution, as then Rmerge > 30%. This results in poor theta_full and theta_max values, as well as poor data/parameters ratio.

Alert level B

```
PLAT340_ALERT_3_B Low Bond Precision on  C-C Bonds ...............    0.01627 Ang.
```

Author Response: Crystals were small and poorly diffracting. Even using a synchrotron source significant diffraction with reasonable completeness was only up to ca. 1.05 angstrom. The present data were cut at 1.10 angstrom resolution, as then Rmerge > 30%. This results in poor theta_full and theta_max values, as well as poor data/parameters ratio, while also probably participating to the relatively low precision on C-C bonds.

Alert level C

```
PLAT042_ALERT_1_C Calc. and Reported MoietyFormula Strings  Differ     Please Check
              Calc: C43 H30 O4
              Rep.: 2(C43 H30 O4)
```

Author Response: There are two, chemically identical, molecules in the asymmetric unit. We prefer to report the corresponding formula with Z=8, instead of the formula with one sole molecule and Z=16.

```
PLAT094_ALERT_2_C Ratio of Maximum / Minimum Residual Density ....       2.58 Report
```

Author Response: At the end of the refinement, there remained one electron density peak of ca. 0.47 in a small void space, while the rest of residual density were > 0.15, resulting in a relatively high Max/Min residual density ratio. An attempts at refining the stronger residual density peak as a lattice water molecule was done, but diverged with Ueq > 0.8. The corresponding void space is therefore likely empty, as confirmed through analysis with PLATON/SQUEEZE.

```
PLAT221_ALERT_2_C Solv./Anion  Resd 2  C   Ueq(max)/Ueq(min) Range        4.7 Ratio
PLAT223_ALERT_4_C Solv./Anion  Resd 2  H   Ueq(max)/Ueq(min) Range        4.7 Ratio
PLAT234_ALERT_4_C Large Hirshfeld Difference C51      --C75       .       0.18 Ang.
PLAT234_ALERT_4_C Large Hirshfeld Difference C57      --C58       .       0.17 Ang.
PLAT234_ALERT_4_C Large Hirshfeld Difference C70      --C71       .       0.16 Ang.
PLAT234_ALERT_4_C Large Hirshfeld Difference C77      --C78       .       0.17 Ang.
PLAT234_ALERT_4_C Large Hirshfeld Difference C79      --C80       .       0.16 Ang.
PLAT234_ALERT_4_C Large Hirshfeld Difference C81      --C82       .       0.18 Ang.
PLAT234_ALERT_4_C Large Hirshfeld Difference C88      --C93       .       0.18 Ang.
PLAT234_ALERT_4_C Large Hirshfeld Difference C92      --C93       .       0.18 Ang.
PLAT234_ALERT_4_C Large Hirshfeld Difference O3       --C6        .       0.18 Ang.
PLAT234_ALERT_4_C Large Hirshfeld Difference C8       --C9        .       0.16 Ang.
PLAT234_ALERT_4_C Large Hirshfeld Difference C17      --C18       .       0.17 Ang.
PLAT234_ALERT_4_C Large Hirshfeld Difference C31      --C32       .       0.19 Ang.
PLAT242_ALERT_2_C Low    'MainMol' Ueq as Compared to Neighbors of          C4 Check
PLAT410_ALERT_2_C Short Intra H...H Contact  H8A      ..H21A      .       1.94 Ang.
                                                      x,y,z  =      1_555 Check
PLAT601_ALERT_2_C Unit Cell Contains Solvent Accessible VOIDS of .          54 Ang**3
```

Author Response: At the end of the refinement, there remained one electron density peak of ca. 0.47 in a small void space, while the rest of residual density were > 0.15, resulting in a relatively high Max/Min residual density ratio. An attempts at refining the stronger residual density peak as a lattice water molecule was done, but diverged with Ueq > 0.8. The corresponding void space is therefore likely empty, as confirmed through analysis with PLATON/SQUEEZE.

```
PLAT910_ALERT_3_C Missing # of FCF Reflection(s) Below Theta(Min).          5 Note
                2  0  0,   0  2  0,   1  2  0,   2  2  0,   0  4  0,
PLAT911_ALERT_3_C Missing FCF Refl Between Thmin & STh/L=    0.457         12 Report
               31 10  0,  27 22  0,  26 24  0,  18 34  0,  30 13  1,  28 19  1,
               25 25  1,   9 39  1,  30  8  2,  28 16  2,  16 31  3,   3 37  3,
```

Alert level G

```
ABSMU01_ALERT_1_G  Calculation of _exptl_absorpt_correction_mu
                not performed for this radiation type.
PLAT003_ALERT_2_G Number of Uiso or Uij Restrained non-H Atoms ...         16 Report
PLAT045_ALERT_1_G Calculated and Reported Z Differ by a Factor ...          2 Check
```

Author Response: There are two, chemically identical, molecules in the asymmetric unit. We prefer to report the corresponding formula with Z=8, instead of the formula with one sole molecule and Z=16.

```
PLAT083_ALERT_2_G SHELXL Second Parameter in WGHT  Unusually Large      11.51 Why ?
PLAT092_ALERT_4_G Check: Wavelength Given is not Cu,Ga,Mo,Ag,In Ka    0.77490 Ang.
PLAT177_ALERT_4_G The CIF-Embedded .res File Contains DELU Records          1 Report
PLAT178_ALERT_4_G The CIF-Embedded .res File Contains SIMU Records          1 Report
PLAT186_ALERT_4_G The CIF-Embedded .res File Contains ISOR Records          1 Report
PLAT188_ALERT_3_G A Non-default SIMU Restraint Value has been used     0.0120 Report
PLAT192_ALERT_3_G A Non-default DELU Restraint Value for First Par     0.0030 Report
PLAT301_ALERT_3_G Main Residue  Disorder .............(Resd    1)         13% Note
PLAT309_ALERT_2_G Single Bonded Oxygen (C-O > 1.3 Ang) ..........          O3 Check
PLAT333_ALERT_2_G Large Aver C6-Ring C-C Dist C81      -C92      .       1.42 Ang.
PLAT410_ALERT_2_G Short Intra H...H Contact  H57A     ..H52D     .       2.05 Ang.
                                                    x,y,z  =      1_555 Check
PLAT860_ALERT_3_G Number of Least-Squares Restraints ............         212 Note
PLAT883_ALERT_1_G No Info/Value for _atom_sites_solution_primary .     Please Do !
PLAT909_ALERT_3_G Percentage of I>2sig(I) Data at Theta(Max) Still        65% Note
PLAT965_ALERT_2_G The SHELXL WEIGHT Optimisation has not Converged     Please Check
PLAT969_ALERT_5_G The 'Henn et al.' R-Factor-gap value ..........       1.80 Note
              Predicted wR2: Based on SigI**2  6.50 or SHELX Weight 12.11
PLAT978_ALERT_2_G Number C-C Bonds with Positive Residual Density.          0 Info
```

```
   3 ALERT level A = Most likely a serious problem - resolve or explain
   1 ALERT level B = A potentially serious problem, consider carefully
  21 ALERT level C = Check. Ensure it is not caused by an omission or oversight
  20 ALERT level G = General information/check it is not something unexpected

   4 ALERT type 1 CIF construction/syntax error, inconsistent or missing data
  12 ALERT type 2 Indicator that the structure model may be wrong or deficient
  11 ALERT type 3 Indicator that the structure quality may be low
  17 ALERT type 4 Improvement, methodology, query or suggestion
   1 ALERT type 5 Informative message, check
```

It is advisable to attempt to resolve as many as possible of the alerts in all categories. Often the minor alerts point to easily fixed oversights, errors and omissions in your CIF or refinement strategy, so attention to these fine details can be worthwhile. In order to resolve some of the more serious problems it may be necessary to carry out additional measurements or structure refinements. However, the purpose of your study may justify the reported deviations and the more serious of these should normally be commented upon in the discussion or experimental section of a paper or in the "special_details" fields of the CIF. checkCIF was carefully designed to identify outliers and unusual parameters, but every test has its limitations and alerts that are not important in a particular case may appear. Conversely, the absence of alerts does not guarantee there are no aspects of the results needing attention. It is up to the individual to critically assess their own results and, if necessary, seek expert advice.

Publication of your CIF in IUCr journals

A basic structural check has been run on your CIF. These basic checks will be run on all CIFs submitted for publication in IUCr journals (*Acta Crystallographica*, *Journal of Applied Crystallography*, *Journal of Synchrotron Radiation*); however, if you intend to submit to *Acta Crystallographica Section C* or *E* or *IUCrData*, you should make sure that full publication checks are run on the final version of your CIF prior to submission.

Publication of your CIF in other journals

Please refer to the *Notes for Authors* of the relevant journal for any special instructions relating to CIF submission.

PLATON version of 06/01/2024; check.def file version of 05/01/2024

Datablock rdt059 - ellipsoid plot

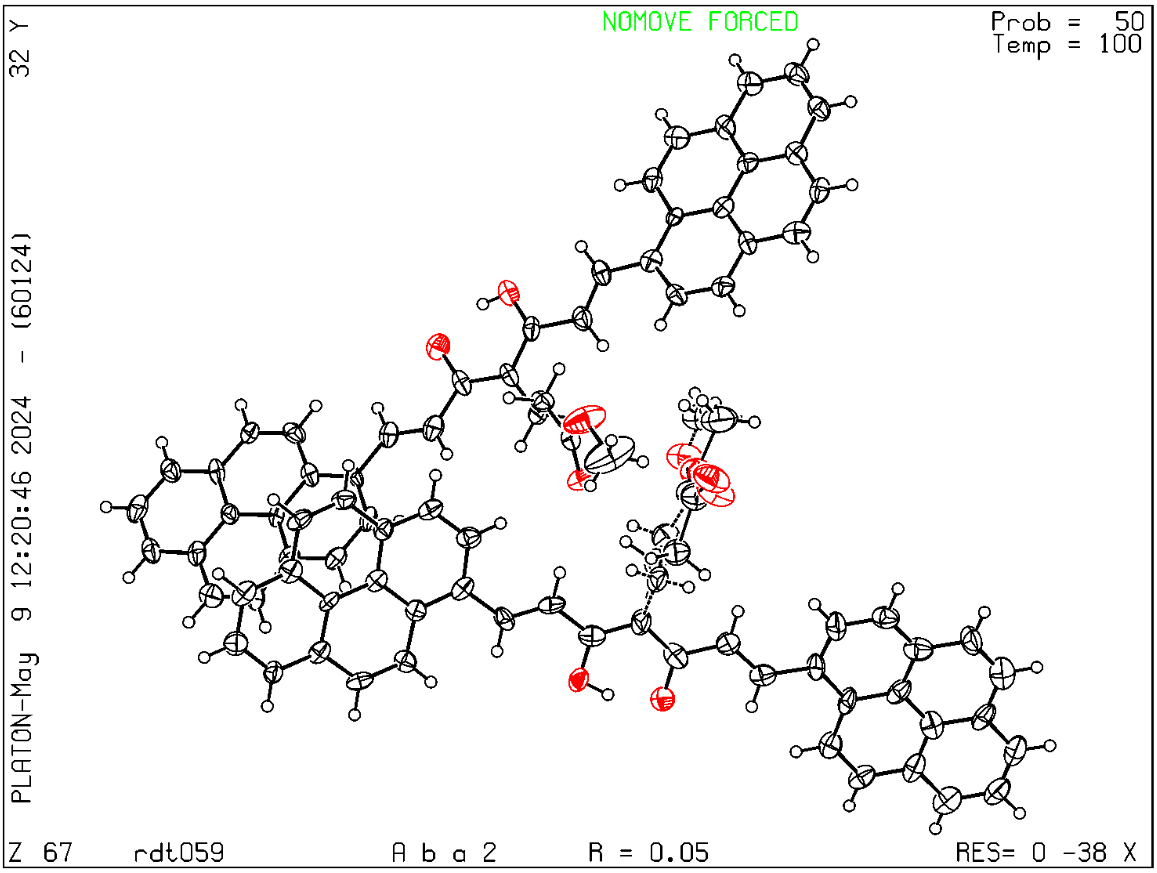

Supplement: Supplementary file 3 — am4c19421_si_003.pdf [file am4c19421_si_003.pdf]
